# Beyond the Definitions of the Phenotypic Complications of Sickle Cell Disease: An Update on Management

**DOI:** 10.1100/2012/949535

**Published:** 2012-08-01

**Authors:** Samir K. Ballas, Muge R. Kesen, Morton F. Goldberg, Gerard A. Lutty, Carlton Dampier, Ifeyinwa Osunkwo, Winfred C. Wang, Carolyn Hoppe, Ward Hagar, Deepika S. Darbari, Punam Malik

**Affiliations:** ^1^Cardeza Foundation and Department of Medicine, Thomas Jefferson University, 1015 Walnut Street, Philadelphia, PA 19107, USA; ^2^Wilmer Ophthalmological Institute, Johns Hopkins Hospital, 400 North Broadway, Baltimore, MD 21267, USA; ^3^Department of Pediatrics, Aflac Cancer Center and Blood Disorders Service, Children's Healthcare of Atlanta, Emory University School of Medicine, Atlanta, GA 30322, USA; ^4^Department of Hematology, St. Jude Children's Research Hospital, Memphis, TN 38105, USA; ^5^Children's Hospital & Research Center at Oakland, 744 52nd Street, Oakland, CA 94609, USA; ^6^Division of Hematology, Children's National Medical Center, 111 Michigan Avenue, N.W., Washington, DC 20010, USA; ^7^Cincinnati Children's Hospital Medical Center, Cincinnati, OH 45229, USA

## Abstract

The sickle hemoglobin is an abnormal hemoglobin due to point mutation (GAG → GTG) in exon 1 of the **β** globin gene resulting in the substitution of glutamic acid by valine at position 6 of the **β** globin polypeptide chain. Although the molecular lesion is a single-point mutation, the sickle gene is pleiotropic in nature causing multiple phenotypic expressions that constitute the various complications of sickle cell disease in general and sickle cell anemia in particular. The disease itself is chronic in nature but many of its complications are acute such as the recurrent acute painful crises (its hallmark), acute chest syndrome, and priapism. These complications vary considerably among patients, in the same patient with time, among countries and with age and sex. To date, there is no well-established consensus among providers on the management of the complications of sickle cell disease due in part to lack of evidence and in part to differences in the experience of providers. It is the aim of this paper to review available current approaches to manage the major complications of sickle cell disease. We hope that this will establish another preliminary forum among providers that may eventually lead the way to better outcomes.

## 1. Introduction

Sickle cell disease (SCD) is an inherited chronic hematological disorder that has no established cure to date except in a few patients who had successful bone marrow or stem cell transplantation. Although gene therapy for sickle cell anemia, the ultimate goal of cure, is not feasible at the present, significant strides have been made at the basic level to achieve the genetic correction of hemoglobinopathies [1]. The molecular lesion of the sickle hemoglobin is a point mutation (GAG → GTG) in exon 1 of the *β* globin gene resulting in the substitution of glutamic acid by valine at position 6 of the *β* globin polypeptide chain [2, 3]. This single-point mutation renders the sickle gene pleiotropic in nature with multiple phenotypic expressions associated with complex genetic interactions and modifiers that are not well understood [2, 3]. The complications of this disease are numerous and affect every organ and/or tissue in the body. Recently concise definitions of these complications have been published [4] thus creating a uniform understanding of the nature of these complications among providers, researchers, patients and their families, and the community at large. The definition of each complication was based on published evidence if available and/or on the experience of experts in the field. The definitions also included the diagnostic criteria, severity index, and classification of each complication whenever available. Specific management and treatment of these complications, however, were not described.

The purpose of this paper is to briefly update the definitions by including newly described complications and review the accepted approaches for the management and treatment of the major complications of sickle cell disease. These will be based on published evidence if available and on the experience of experts in the field. To that end management of pain syndromes, hematological, neurological, ophthalmological, pulmonary, hepatobiliary, splenic, renal, genitourinary, musculoskeletal, and dermatological complications will be addressed. Recently, there has been increasing evidence that asthma predisposes to certain complications of sickle cell disease including acute painful crises, acute chest syndrome, pulmonary hypertension and stroke [5]. Management of comorbid conditions, however, will not be addressed except in certain situations where the comorbid condition has a direct effect on the manifestation and management of the sickle cell complication in question. It is hoped that that this paper together with the previously published definitions will together constitute a review of the state of the art on the complications of SCD and their management.

### 1.1. Recently Reported Complications

#### 1.1.1. Neurocognitive Impairment

Neurocognitive impairment [4, 6] is an invisible complication of sickle cell anemia (SS) that defies detection by imaging and other routine diagnostic methods. Impaired neurocognitive function in seemingly neurologically intact patients is not related to vasoocclusion or hemolysis. It is detected by neuropsychiatric and neurobehavioral testing and is associated with anemia and age.

A controlled cross-sectional multicenter study [6] compared the neuropsychological function and neuroimaging data from 150 adult patients of African descent with SS who had no neurological symptoms with 52 community control adults of African descent with Hb AA. The affected patients and controls were stratified by age and sex. The patients with SS were anemic (hemoglobin levels <10 g/dL), whereas the controls had normal hemoglobin levels. The primary outcome of the study, mean nonverbal function assessed by the Wechsler Adult Intelligence Scale III Performance IQ Index, was significantly lower in patients than controls (86.69 versus 95.19). Significant differences were also noted in secondary measures including global cognitive function, working memory, processing speed, and executive function. Moreover, anemia was associated with worse neurocognitive function in older patients. However, volumetric magnetic resonance imaging measurements showed no significant differences in total gray matter or volume of the hippocampus between patients and controls, but there was a nonsignificant reduction in brain volumes with older age among adults with SS. Although lacunae were more frequent in patients with SS, these lesions were not related to neurocognitive function.

Another uncontrolled study from a single institution [7] showed that 11 adult patients with SCD had neurobehavioral impairment independent of neuroimaging abnormalities. Moreover, other studies [8, 9] reported neurocognitive impairment in children with remote silent cerebral infarcts (SCIs). Thus it is not clear whether neurocognitive impairment is due to the anemia or to the infarct or both. Studies are underway to determine if blood transfusion will improve neurocognitive function of patients with and without SCI. Other investigators [10], however, observed that regular chronic blood transfusion does not prevent CSI or reverse cerebral vasculopathy especially in patients with a history of overt stroke.

#### 1.1.2. Acute Silent Cerebral Infarcts (SCIs)

The term “Acute Silent” seems paradoxical since acuity is usually associated with overt signs and symptoms whereas silence is associated with covert findings. Nevertheless, in an observational study [11] pediatric hematologists reviewed their experience with acute SCI detected by diffusion-weighted MRI (DWI) in children with SS. Their conclusion suggests that there is a sequence of events that starts with an acute SCI that later evolves into remote SCI. The acute event is most often precipitated by severe anemia due to exacerbation of the steady-state values. The severe anemia is associated with or initiates cytotoxic edema in the brain which is visualized on DWI as a hyperintense punctuate area of restricted diffusion that corresponds to a decreased signal on the apparent diffusion coefficient (ADC) map. The DWI abnormal signal appears within 24 hours of stroke onset and persists for 10–14 days only. Later on, the usual T_2_-weighted abnormality from the stroke develops and persists indefinitely as a remote SCI. It is not clear how long it takes before the permanent abnormality appears.

### 1.2. General Principles of Management of the Complications of SCD

The complications of SCD are divided into three major types: (1) pain syndromes and related issues; (2) anemia and its sequelae; (3) organ or tissues damage. Management of these complications will be described below. Management of SCD in general and its complications in particular follow five major approaches (Table 1). These include supportive management, symptomatic treatment, preventative management, and abortive and curative approaches to management. Although these approaches apply to the disease as a whole, at least one of them applies to each complication as will be discussed below.

## 2. Management of Specific Complications

### 2.1. Management of Acute Anemia in SCD

The majority of patients with sickle cell disease (SCD) have some degree of baseline anemia due to ongoing hemolysis. Although there is interpatient variability, individuals with hemoglobin SC and sickle *β* ^+^ thalassemia generally have less severe anemia than hemoglobin SS and sickle *β* ^0^ thalassemia. Certain SCD-related complications, such as splenic sequestration, aplastic crisis, and hyperhemolytic crisis, can lead to acute exacerbation of anemia [12]. Below, we briefly describe causes of acute anemia and discuss management of these complications.

#### 2.1.1. Acute Splenic Sequestration

The spleen undergoes variations in size during childhood in SCD. It may be initially enlarged in children with SCD but may become dysfunctional as early as in first year of life. More than 90% of children with sickle cell anemia (SCA) may have total loss of functional splenic tissue by early childhood [13, 14]. Children with SCA who have not yet gone through autosplenectomy, as well as SC disease and sickle beta thalassemia, may be at risk for developing splenic sequestration. Hemoglobin F likely plays a role in rate of spleen atrophy; as a result individuals with high fetal hemoglobin concentration retain splenic function longer than those with lower fetal hemoglobin and remain susceptible to splenic sequestration. Most cases of acute splenic sequestration occur between 3 months to 5 years of age, but it has been reported in infants as young as 5 weeks [15] as well as in adults [16]. Acute splenic sequestration is a significant cause of morbidity and could be fatal in a few hours if left untreated, especially in children [17]. Episodes of splenic sequestration in adults are generally mild and have been reported in patients with hemoglobin SC disease and sickle *β* ^+^ thalassemia at risk due to occasional persistent splenomegaly into adulthood [18]. Early parental education to identify splenic sequestration and seek urgent medical attention in the event of symptoms of enlarging spleen has been successful in reducing mortality associated with this complication in SCD children [19].

Acute splenic sequestration in SCD results from the trapping of red cells in the splenic sinuses which leads to a sudden rapid enlargement of the spleen which could be massive. These episodes are generally associated with viral or bacterial infections. Patients present with pallor, tachycardia, tachypnea, weakness, abdominal pain and distension, and shock due to hypovolemia and acute decline in hemoglobin level. Mild thrombocytopenia may also be present. The cooperative study of sickle cell disease (CSSCD) defined acute splenic sequestration as decrease of hemoglobin or packed cell volume (PCV) of at least 20% from the baseline along with increase in palpable spleen size of at least 2 centimeters from baseline [20]. Hepatic sequestration, which is characterized by tender hepatomegaly, acute exacerbation of anemia, reticulocytosis, and hyperbilirubinemia, can also occur, though more rarely [21]. However, due to the limited capacity of the liver to expand, hepatic sequestration is generally not associated with cardiovascular collapse.

Acute splenic sequestration should be managed with urgency due to its potential life-threatening nature. Immediate treatment of acute splenic sequestration includes the correction of hypovolemia to avoid hypovolemic shock and the transfusion of packed red cells to maintain oxygen-carrying capacity. Once the cardiovascular status is restored, the patient improves rapidly and the spleen shrinks in few days releasing the trapped red cell back into circulation. Hemoglobin level increases often to a greater extent than the predicted based on the red cell volume administered. Therefore, to avoid hyperviscosity and related complications, patients should not be transfused to achieve baseline hemoglobin levels.

The rate of recurrence of an acute life-threatening episode of splenic sequestration is high, occurring in approximately 50% of those who survive first episode [22]. To eliminate recurrence some have recommended elective splenectomy after first episode [23] while others citing concerns for postsplenectomy sepsis have suggested splenectomy after two episodes of sequestration [24]. However in situations when there is concern that child may not be able to reach medical facility in time, splenectomy should be strongly considered after the first episode of splenic sequestration. Emergency splenectomy during an episode of acute sequestration is not recommended. Partial splenectomy has been considered as an approach for preventing recurrences while retaining splenic function [25, 26]; however this approach is much less standard, and overwhelming sepsis has been described in patient who underwent partial splenectomy [27]. Since splenectomy may increase the risk of invasive sepsis, all postsplenectomy patients should be vaccinated against pneumococcus and meningococcus and should receive lifelong penicillin prophylaxis. Limited-term red cell transfusion has been recommended as a strategy to delay splenectomy in young children under age 3 years [28]. While one study deemed short-term transfusion beneficial in reducing the risk of splenic sequestration and temporarily reversing splenic dysfunction [29], another study found it of limited value [30]. When considering chronic red cell transfusion, the goal should be to maintain hemoglobin S level less than 30%. Observation following an episode of sequestration occasionally may be a reasonable strategy such as in SCD adults in whom these episodes are generally milder.

#### 2.1.2. Acute Aplastic Crisis

In a steady state of health in SCD, shortened lifespan of red cells remains compensated by an increased production of erythroid cells by the bone marrow. Thus even a temporary cessation of erythropoiesis in SCD can lead to acute exacerbation of anemia. Suppression of erythropoiesis can be caused by many infections, including the classic example of parvovirus B19, which preferentially attack erythroid precursors due to P-antigen of erythrocyte being its receptor. Destruction of erythroid precursors leads to severe anemia and reticulocytopenia [31].

The clinical course following Parvovirus B19 infection is variable, and not all patients develop severe anemia. Episodes of aplastic crisis are generally preceded by febrile illness. Patients present with fatigue, pallor, and weakness along with laboratory evidence of anemia and reticulocytopenia (usually <1%) [4, 12]. Recovery generally occurs in a week. During the recovery phase, patients develop brisk reticulocytosis, which combined with anemia, may be misdiagnosed as hyperhemolytic crisis. In patients presenting with red cell aplasia serologic samples to confirm the Parvovirus B19 infection should be sent. Parvovirus B19 has been described to be associated with other complications of SCD including acute splenic sequestration [32], encephalopathy [33], and acute chest syndrome [34]. Lifelong immunity develops following a Parvovirus infection. Although many patients recover from the transient erythroid aplasia spontaneously, packed red cell transfusion should be considered for symptomatic patients. The secondary attack rate of siblings with SCD is high, so they should also be monitored closely for development of aplastic crises. Additionally, patients suspected to have Parvovirus infection should be isolated from pregnant staff because Parvovirus infection during pregnancy can lead to hydrops fetalis [35].

#### 2.1.3. Hyperhemolytic Crisis

Hyperhemolytic crisis is defined as the presence of acute anemia along with the evidence of accelerated hemolysis. It presents with acute reduction in hemoglobin level often associated with reticulocyte count that is higher than the baseline. Several subphenotypes have been described including hyperhemolysis during an episode of acute vasoocclusive painful crises [36] or as an acute or delayed hemolytic reaction following a transfusion of red cells [37, 38]. Acute hemolysis can also occur in context of infection such as malaria or drug exposure. Management is dependent on the cause. Investigations should include evaluation to rule out autoimmune hemolysis presenting as acute or chronic hemolytic transfusion reaction where both autologous and transfused red cells are destroyed [37]. Alloimmunization increases the risk for hemolytic transfusion reactions. SCD patients have higher incidence of RBC alloantibodies with one reason being differences in the frequency of antigen distribution between largely Caucasian donor pool and SCD patients who are of African ancestry [28]. CSSCD reported overall rate of alloimmunization to erythrocyte antigens at 18.6% [39]. Risk of alloimmunization increases with number of transfusions received by the patient [39].

Hyperhemolysis associated with painful crises can often be managed conservatively, but transfusion of red cells can be considered if clinically indicated. A high index of suspicion is needed in case of transfusion-related hyperhemolysis, because symptoms may be similar to painful crises including bone pain and fever making the identification difficult. It is a serious and potentially life-threatening complication of red cell transfusion. The laboratory evaluation in delayed hemolytic transfusion reaction (DHTR) may reveal low hemoglobin, elevated serum lactate dehydrogenase, and bilirubin above the patient's baseline. Serial hemoglobin levels, reticulocyte counts, and hemoglobin electrophoresis may be helpful in making a diagnosis of hyperhemolysis in the posttransfusion setting. DHTR is often associated with a positive antiglobulin test (DAT) [40]. The management of posttransfusion hyperhemolysis is generally supportive. Further transfusions should be avoided, as they often lead to further hemolysis. However subsequent further transfusions may be needed if hemolysis is rapid and severe. Additional transfusions with corticosteroids and intravenous gamma globulin have been successfully used in severe cases of post transfusion hyperhemolysis [41–43]. Use of rituximab for prevention of delayed hemolytic transfusion reaction has also been reported in a patient with SCD [44].

## 3. Gastrointestinal/Hepatobiliary Complications

Sickle cell disease affects the hepatobiliary system in different ways at different ages. Intrinsic disease results from recurrent ischemia and bilirubin stones. These result from the vascular obstruction and red cell hemolysis of sickle cell. Biliary sludge is a common finding that is often clinically unimportant. Viral infections that affect the liver may be independent of or secondary to red cell transfusions. The iron overload that accompanies red cell transfusions can lead to liver dysfunction and fibrosis. Many medications taken by sickle cell patients may cause or worsen hepatobiliary disease. The dysfunction of the liver can affect the lungs, kidneys, and coagulation systems. Treatment is directed at the etiology of the dysfunction as well as the underlying sickle cell disease.

The natural consequences of any hemolytic condition affect both the gallbladder [45] and the liver [46]. The gallbladder is affected by hemoglobin (pigmented) stones [47], biliary sludge [48–50], and obstruction [51–53]. The liver is affected by vasoocclusive changes (right upper quadrant syndrome) of recurrent ischemia and reperfusion injuries [46, 54], iron overload from transfusions that are used to treat both symptomatic anemia and the complications of sickle cell disease [55–59], vascular endothelial dysfunction [60], and the liver consequences of the hypercoagulation of sickle cell [61–63].

The challenge physicians caring for sickle cell patients is recognizing the life-threatening course from the more frequent, similar appearing milder, recurrent syndromes. A useful way to consider the protean effects of hepatobiliary issues in sickle cell is to consider the disorders of the presentation and evaluation of abdominal complaints of sickle cell followed by a review of the major disorders. Although hepatobiliary conditions are intimately linked, the embryology of the biliary system and the hepatic system shows these two organs to be histologically and functionally separate [64]. This explains the differential response of these organs to the same insult. However, many conditions may overlap, so a single diagnosis may mask parallel processes.

### 3.1. Right Upper Quadrant Syndrome

Acute pain in the right upper quadrant is common in sickle cell patients [65–67]. The symptom of hepatobiliary disease often must be separated from the more common symptoms of sickle cell disease. Patients develop sickle cell attacks in a consistent pattern. The patient can often recognize whether the current attack is different from prior sickle cell pains. If the pain is new, especially when accompanied by more jaundice than usual, nausea and vomiting, then further hepatobiliary workup is needed. Increasing nausea and vomiting with food points to the gallbladder. Colic pains point to the gallbladder. Right upper quadrant fullness with dull pains points to the liver. General jaundice points to both.

### 3.2. Hepatomegaly and Ischemic Changes Are Common

The liver is often increased in size throughout the life of the patient [68]. If the liver has acutely increased in size, then hepatic congestion or sequestration may be involved. A 1980 clinicopathologic study of 70 autopsies of sickle cell patients found 91% with enlarged livers characterized by distention of Kupffer cells engorged with red cells [69]. In 27% the liver sinusoids were distended with obstruction from sickled red cells. Focal necrosis of liver tissue was present in 34%. 20% of patients had reparative liver changes of portal fibrosis and regenerative nodules. The authors felt that recurrent vascular obstruction, ischemia, necrosis, and repair best explained the pathological findings.

### 3.3. Diagnostic Clues

If right upper quadrant pain is severe, then acute swelling or inflammation may be involved. Murphy's sign is often lost in the general pains but, if present, may point to the gallbladder. If the serum bilirubin concentration is over 4 mg/dL, then checking whether the fraction of direct bilirubin exceeds 10% would point to the gallbladder as the source of the increase [70, 71]. Some patients have genetic variations in the UDP glucuronyltransferase that will elevate the serum bilirubin concentration [72]. This recurrent or chronic elevation should be evident on review of the patient's records. In most sickle cell presentations the AST is relatively more elevated that the ALT, as the AST also reflects the degree of hemolysis [73]. If the ALT is similarly elevated as the AST, then a hepatocellular process may be occurring. Similarly the alkaline phosphatase will be elevated in biliary disease. However, bone infarcts will also call the alkaline phosphatase to rise. Fractionating the alkaline phosphatase into bone and biliary sources is seldom done. The clinical presentation usually finds bone pain or severe extremity pains with infarcts, and severe right upper quadrant pains prompt imaging, usually ultrasound, of the hepatobiliary system. Measurement of the aPPT and PT may provide evidence of a more severe process beginning.

### 3.4. Key Points to Remember

- Initial evaluation is for conditions that need emergent transfusions or treatments.
- Pain patterns that differ from a patient's usual pattern need close evaluation.
- Having sickle cell does not protect a patient from any other condition.
- Liver involvement may be part of a multiorgan failure syndrome [74, 75].
- With any severe sickle cell complication, exchange transfusions are often the treatment of choice [76–79].

*Hepatic crisis* is often used as a general term to describe right upper quadrant pain in a sickle cell patient [80, 81]. However, hepatic crisis is best used to describe a syndrome consisting of pain, elevated ALT (usually less than 300 IU/liter), and hepatic enlargement. Another working definition of a hepatic crisis could be painful hepatomegaly and worsened jaundice (usually less than 12 mg/dL) [82]. The definition used causes the incidence of this condition to vary in reports. Large series reports that up to 10% of patients admitted to hospital have hepatic involvement rising to their definition of crisis. Other studies with more restrictive definitions concluded hepatic crisis was rare. The rapidity of the onset of symptoms and the rapidity of the correction of ALT may be able to guide therapy. Symptoms that began suddenly are more often typical, self-limited sickle cell conditions. Symptoms that begin over several days to weeks may be from more severe conditions such as viral or autoimmune hepatitis, liver infarct, or gallbladder dysfunction. Severe elevations of bilirubin (over 30 mg/dL) may represent acute liver failure of intrahepatic cholestasis (see below).

If the condition is from typical sickle vaso-occlusion and inflammation, then the elevation of ALT decreases after a few days. Severe, persistent elevations may relate to hepatic infarct, characterized by a wedge-shaped, hypointense CT lesions [83]. Hepatic abscess has been rarely reported, but should be suspected in a patient with fever, a course different from their usual sickle cell crisis, right upper quadrant pain, and tender hepatomegaly [84–88]. Hepatic ultrasound would delineate the abscess. Prior areas of hepatic infarction give the bacteria a site to invade. *Bacteroides* species were found in one report [85]. Bilirubin levels decrease to prior values in about two weeks; liver transaminases return to prior values in about three months. If changes persist beyond those times, further evaluation is needed.

*Hepatic sequestration* is best diagnosed by a rapid enlargement of the liver with a concurrent drop in hemoglobin concentration [89–91]. The bilirubin also will be elevated with a high percentage of direct bilirubin. Transfusions, simple or exchange, may help reserve the process. Hepatic sequestration may be a life-threatening event in pediatric patients with sickle cell disease [89–91]. Small vessel congestion with red cells leads to a drop in hemoglobin levels. The liver enlarges and becomes tender and inflamed. Treatment is transfusions. Often the hemoglobin level is low enough that given red cell units (matched for ABO, Kell, E, and C antigens) to raise the hemoglobin to 9 g/dL often stabilize the process. Manual or automatic red cell exchanges are indicated for more severe cases shown by hepatic dysfunction or a hemoglobin level over 9 to start with. Hepatic sequestration may be part of the multiorgan failure syndromes [74, 75].

Chronic hepatic sequestration has been reported in a 17-year old with SS hemoglobin [92]. After exchange transfusions, his liver size decreased. However it recurred. This recurrence was successfully treated with hydroxyurea for several months.

One report of “reverse sequestration” occurred following simple transfusions. This syndrome comprises a sudden increase in hemoglobin concentration, sudden onset of hypertension, acute congestive heart failure, neurologic signs of infarct or hemorrhage [93].

*Autoimmune hepatitis* is reported in sickle cell patients [94, 95]. Interestingly, it also appears in mice models of sickle cell disease (personal communication). We have documented transient positivity of antibodies to smooth muscle (antiactin F). Associated features of autoimmune hepatitis include rashes, skin ulcers, and joint disease. The etiology, natural course, and treatment of autoimmune hepatitis in sickle cell patients are unclear. If a patient has persistent liver symptoms and antibody titers to smooth muscles, then a therapeutic trial of prednisone and azathioprine may be warranted. Referral to a hepatologist is indicated.

*Viral hepatitis* occurs at least as frequently as in the general population [96]. Hepatitis C, and to a lesser extent, Hepatitis B, occurred more often because of blood product exposure. Improved blood product testing has reduced the incidence of these infections, but they still occur. We screen all our patients yearly for Hepatitis C viral RNA by PCR. In new patients, persistently elevated ALT levels require screening for viral hepatitis. Every sickle cell patient should be vaccinated with two doses of Hepatitis A vaccine from six months to a year apart and three doses of Hepatitis B vaccinations at zero, one, and six months. Quantitative hepatitis B surface antibody tests and total Hepatitis A antibody tests are available to help decide if a patient has been adequately vaccinated if the records are not available. Many practitioners opt to revaccinate in case of any doubt. No vaccine exists for Hepatitis C prevention. Patients with chronic Hepatitis B and Hepatitis C should be treated as any other patients. There has been some concern about using ribavirin because it may cause hemolytic anemia. If a patient on ribavirin does develop worsening anemia, then placing the patient on monthly transfusions would both allow therapy to continue and would decrease sickle cell and anemia symptoms. A recent article showed good results in treating sickle cell patients for chronic hepatitis C [97]. Liver transplants are as successful in patients with sickle cell disease and other patients needing allographic livers [98–101].

*Hepatic siderosis* is a growing area of concern and research [102]. As red cell transfusions become routine for more indications, the inevitable result is the accumulation of liver iron. After about a year of transfusion therapy, serum ferritin levels rise to over 1,000 ng/mL. While serum ferritin is a rough guide to total liver iron, values over 1,000 indicate liver iron overload. Other studies have shown significant liver iron accumulation after 13 units of red cells. Each unit of red cells contains nearly a year worth of dietary iron. Over many years, hepatic dysfunction, insufficiency, fibrosis, and cirrhosis may lead to morbidity and even liver death. Many patients on regular transfusions will have hyperintense livers on CT scans or hypointense livers on MRI scanning [103, 104]. These changes have been used to semiquantitate the degree of iron loading. Chelation with deferoxamine [55, 105], deferasirox [106], or deferiprone (recently approved in the US) does reduce total body iron. However, all regimes have issues with compliance and side effects that require appropriate monitoring. When patients with iron overload are admitted to hospital with noninfectious complaints, we often give deferoxamine 3 grams in 500 mL normal saline intravenously over 24 hours, repeating continuously during their stay. Giving Vitamin C 250 mg orally daily while the patient is on deferoxamine increases iron excretion [107, 108]. Ongoing cohort studies should help define the natural history of iron overload in sickle cell patients [109–111].

*Hepatic effects on kidneys and lungs* are increasingly recognized. Although there are few publications concerning sickle cell patients, such effects are well known in other conditions where the liver is cirrhotic or dysfunctional. The hepatorenal syndrome [112], hepatopulmonary syndrome [113], and the portopulmonary [114] syndrome may complicate the hepatic disease of sickle cell.

*Sickle cell intrahepatic cholestasis* or *sickle cell hepatopathy* is a condition with marked hyperbilirubinemia (>50 mg/dL) and a high fraction of direct (conjugated) bilirubin (about 50%) [77, 115–118]. Other features of right upper quadrant pain and progressive hepatomegaly resemble many of the hepatic crisis syndromes. However, in sickle cell intrahepatic cholestasis, the liver transaminases are nearly at baseline. Coagulopathy as assessed by the PT test is often found. Renal insufficiency is often present, likely from the nephrotoxic effects of bilirubin. Endoscope retrograde cholangiopancreatography has been reported to guide management by diagnosing strictures from ischemic cholangiopathy and defining the presence or absence of common bile duct stones [119]. Some authors consider the presence of acute sickle hepatopathy to contraindicate liver biopsies [120]. Ischemic cholangiopathy has also been described [121].

Early reports indicate that sickle cell intrahepatic cholestasis was a life-threatening condition that mandated exchange transfusions. As clinicians were more aware of the condition, series were reported that had a less severe course [122]. Given the protean causes of intrahepatic cholestasis, it is reasonable to divide cases of cholestasis into those with and those without other evidence of marked hepatic dysfunction and coagulopathy. The milder cases (bilirubin level 10 to 30 mg/dL) appear to be more common in children. Patients in the first category should be monitored for worsening hepatic function: encephalopathy, coagulopathy, and rising bilirubin concentrations. For the more severe cases, exchange transfusion may be given, but it is not always effective [77, 79].

*Cholelithiasis* occurs as early as two years old [47]. About 30% of patients will have gallstones by 18 years of age [52, 123, 124]. The incidence and prevalence of this condition appears to be affected by local diet and possible genetic factors [125]. The coinheritance of *α*-thalassemia may reduce the incidence of stones since it may lessen the degree of hemolysis that is thought to drive stone formation [126]. The cause of cholelithiasis is usually pigmented stones resulting from the breakdown of hemoglobin [45]. Some reports implicate ceftriaxone and other third generation cephalosporins as causing crystallization in the gallbladder [127]. However, these antibiotics are commonly and usefully used in the proper settings. In adults, asymptomatic gallstones are common and are best treated by observation only [52, 53, 68, 123]. Abdominal and right upper quadrant pains are common in sickle cell patients. Cholecystectomy for recurrent right upper quadrant pains often does not relieve the recurrent symptoms. Only if signs of cholecystitis (fever, increased direct bilirubin, and positive imaging) develop, should cholecystectomy be considered after the treatment with supportive care and antibiotics [47, 124]. Laparoscopic cholecystectomy is the procedure of choice for this indication [128, 129]. This also causes less abdominal muscle disruption and decreases postsurgical complications including acute chest syndrome. Ultrasound is the imaging of choice but is not diagnostic in most cases. Reports of pancreatitis from sickling also exist. Biliary scintigraphy is seldom used because of the numerous false positive results [130, 131]. Still, it has a useful negative predictive value if used in the right setting. Technetium scanning may show hyperemia of cholecystitis but its use is not well studied. Liver peliosis and extramedullary erythropoiesis have occasionally been noted as multiple nodules on liver imaging [132].

*Biliary sludge* is a common finding in sickle cell patients [48, 50]. Biliary sludge is nonshadowing, echogenic intraluminal sediment. This material is calcium bilirubinate, cholesterol crystals, viscous bile, mucus, and proteins. The natural history of biliary sludge in children with sickle cell disease finds that at a mean of 2.1 years of followup, about 65% of such patients do eventually develop gallstones, although not necessarily symptomatic ones. About 40% of patients originally with biliary sludge do not develop gallstones, despite the continued presence of sludge in most [133]. Most authors recommend yearly ultrasounds to access stone formation. They reserve cholecystectomy only for patients with signs and symptoms of acute cholecystitis [133].

*Choledocholithiasis* also occurs in sickle cell disease [51]. Even in patients with cholecystectomy, recurrent stones may form in the common bile ducts. Symptoms are similar to primary gallbladder disease. Ultrasound may be the best modality to evaluate the common bile duct. Duct obstruction is seldom complete. This may be because pigmented stones are smaller than nonpigmented stones. If the common duct is obstructed, then symptomatic or chemical pancreatitis may be the presentation [134]. After cholecystectomy, the common bile duct is usually dilated, confounding diagnosis of new stones. Given the prevalence of common duct stones, patients with persistent cholestatic jaundice should have imaging to evaluate the ductal system. If surgery is contemplated, some authors suggest ERCP as the best approach to determine management [135].

*Acute cholecystitis* presents as it does in patients without sickle cell disease [53, 136]. Right upper quadrant pain, fever, nausea, and vomiting have a long and diverse differential diagnosis. When the diagnosis is suspected, then ultrasound is the usual next step. Imaging signs of acute inflammation or obstructing stones prompt treatment for pain, hydration, and the assessment for infection. Laparoscopic cholecystectomy is deferred until the acute episode is over. If all the stones and sludge have cleared, then surgery may not be indicated. Some authors prefer a conservative approach. Intraoperative cholangiography is reported to have a 25% false positive rate. Some authors recommend intraoperative ERCP. A detailed intraoperative evaluation of the biliary system is important as symptoms often persist or recur after cholecystectomy [124].

*Chronic cholecystitis* may be related to persistent gallstones or persistent biliary sludge. Recurrent symptoms consistent with colic warrant screening with blood work and imaging. If the blood work shows increases in conjugated (direct) bilirubin during the attacks, and there are ultrasonographic signs of a thickened gallbladder wall, then cholecystectomy may decrease these symptoms. However, just as in chronic cholecystitis in the general population, the symptoms may recur several months after surgery.

### 3.5. Summary

Disorders of the hepatobiliary symptoms are common in sickle cell disease. Besides the conditions found in the general population, several conditions occur that are specific to sickle cell disease. These conditions include the prevalence of pigmented stones, intrahepatic cholestasis, hepatic sequestration, and recurrent hepatic ischemia and necrosis. A directed history and physical examination will suggest needed further evaluations. Laboratory values and imaging will often establish the diagnosis. Cholecystectomy is best reserved for symptomatic patients. Biliary sludge should be followed for the development of symptoms. For any severe or multiorgan dysfunction in sickle cell patients, red cell transfusions or exchange transfusions will stabilize the underlying sickle cell disease and may reverse the pathologic process.

## 4. Muscular/Skeletal/Skin Complications

### 4.1. Dactylitis

Dactylitis is usually the earliest musculoskeletal manifestation of SCD and occurs in infants and very young children with a peak incidence during the first 6–12 months of life [137]. Prevalence rates of dactylitis are roughly 45% before age 2 years. It occurs more often during cold seasons and is associated with a lower fetal hemoglobin and higher reticulocyte counts [138]. In the CSSCD study [139, 140] similar to the Pediatric Cohort of Guadeloupe 1984–1999 [141] dactylitis particularly occurring prior to age 6 months was a predictor of adverse outcomes including death, ACS, stroke, and frequent pain [142]; however this was not reproduced in the Dallas cohort, and dactylitis has limited utility as a predictor of outcome [143]. Ischemia/infarction of the bone and marrow is associated with increased erythropoiesis and bone marrow expansion involving the hands and feet and results in tenderness, swelling, redness, and warmth of the affected limb/digit. Bony destruction of the terminal phalanges and metacarpals may occur from prolonged ischemia and or superimposed osteomyelitis. The administration of malaria prophylaxis was found to reduce the number of episodes of both malaria and dactylitis [144]. The mainstay of treatment for dactylitis remains oral or parenteral nonsteroidal anti-inflammatory agents and aggressive intravenous hydration. Opioid analgesics provide additional pain control as needed, and topical warm packs may help ease discomfort and swelling. Attention to treating coexisting infection and ruling out secondary osteomyelitis of affected bones is prudent. In the recently reported BABY HUG randomized controlled trial, the use of hydroxycarbamide resulted in a significant reduction in the rates of dactylitis in very young children with SCD (24 events in 14 patients versus 123 events in 42 patients in the placebo group, *P* > 0.0001) [145].

### 4.2. Osteopenia/Osteoporosis

Osteoporosis (OP) and low bone mineral density (BMD) or osteopenia is now being recognized as a common bone complication in both children and adults with SCD. The prevalence of low BMD in SCD ranges from 30 to 80% [146–151] with a predilection for the lumbar spine. Increased hemolysis, (low hemoglobin, high LDH, high reticulocyte count), hemoglobin F, age, sex hormone status, number of vaso-occlusive events, and body mass index (BMI) have all been correlated with BMD in SCD [152–155]. Often a normal BMD at the femoral neck, particularly in a patient with avascular necrosis may give false reassurance representing local increase in bone remodeling in response to infarction and necrosis. Fractures involving the long bones and spine are grossly underdiagnosed in SCD in part due to the high rates of pain from other more common etiologies (acute vaso-occlusion, bone infarcts, osteonecrosis, and chronic marrow expansion). Thus the expected relative risk of fractures based on the high prevalence rates of OP in SCD has not been documented. Only one published report compares fracture rates among persons with SCD and thalassemia patients and showed similar fracture rates for SCD and the general population [156]. This raises the question as to whether the clinical outcomes of OP may be modulated by the vaso-occlusive phenomena seen with SCD. Many times, radiographs are not obtained with exacerbation of pain symptoms as it is assumed to be typical vaso-occlusive pain that is characteristic of the disease. Also, both clinicians and radiologists are usually underwhelmed by the presence of so-called sickle cell bony changes on plain radiograph as these are seen almost universally making it easy to overlook subacute and chronic fractures or osteopenia.

The specific etiology of osteoporosis in SCD is multifactorial with many similarities to the OP found in thalassemia syndromes albeit less well studied [157, 158]. Hypogonadism (delayed puberty and/or secondary hypogonadism associated with hemosiderosis) is a well-recognized cause of OP and is associated with increased bone turnover [159]. Delayed constitutional growth and maturation associated with IGF-1 deficiency, hypothyroidism as well as micronutrient deficiencies are also commonly seen in SCD and negatively impact optimal bone mass accrual [160, 161]. Bone marrow expansion from chronic anemia and increased erythropoiesis, increased bone turnover from vitamin D deficiency, recurrent bone infarcts and vaso-occlusion, chronic inflammation as well as sedentary lifestyle due to pain all contribute to development of osteoporosis in SCD [162–166]. Iron overload has both direct and indirect effects on bone density. Directly, increased iron deposition in bone marrow leads to chronic inflammation, inhibition of osteoblast function, and increased osteoclast activity leading to bone resorption, cortical and trabecular bone abnormalities [167–169]. Iron overload by its effect on endocrine organs leads to hypogonadism, hypopituitarism, and ultimately reduced BMD [155, 156, 160, 161, 164, 165, 170]. Genetic determinants of BMD have been investigated in several populations and groups but are not well studied in SCD. Polymorphisms of the vitamin D receptor (VDR) and collagen type I alpha 1 gene (COLIA1) have been associated with reduced BMD in postmenopausal and thalassemia and predispose women to osteoporotic fractures [171–174]. These correlations, however, have not been confirmed in SCD.

According to the World Health Organization (WHO) to be diagnosed with osteoporosis, one must have a bone mineral density (BMD or bone mass) of at least 2.5 standard deviations (T score) below the peak bone mass typically achieved by a healthy adult between the ages of 18–30 years of age of the same sex and ethnicity [175] (WHO Scientific Group on Prevention and Management of Osteoporosis 2000: Geneva, Switzerland, 2003. “Prevention and management of osteoporosis: report of a WHO scientific group”). A z-score is used to report BMD that is corrected for age as well as sex and ethnicity; therefore it is more applicable to pediatric subjects. Regardless of definition, the high rates of low BMD in SCD predispose to fractures and deterioration of bone; however a consensus on how to manage low BMD in SCD currently does not exist.

The implications of low BMD to the aging patient with SCD are far reaching. Osteoporosis is frequently asymptomatic; when fractures occur, they cause significant, morbidity including pain, deformities and vertebral collapse [176]. Requirement of long-term analgesia, mechanical support, and surgical interventions increases exponentially following OP-related bone complications. The literature on fracture prevalence in SCD is however very limited [156, 177–179]. A study by Fung et al. found the self-report rates of fracture among young adults (median age 25 years) with SCD to be 32% and 28% for nontransfused and transfused males and 15% and 16% for nontransfused and transfused females, respectively [156]. In this report, most fractures occurred in the upper extremities (47.4%), lower extremity (29.5%), or spine and pelvis (11.5%), and fracture rates increased with age. Ebong reported fracture rates of 20% in persons with SCD and osteomyelitis, the majority of whom were children [179]. As the natural history of SCD continues to evolve, the prevalence of age-related diseases such as OP will be better known. This is significant because hip fractures are associated with a one-year mortality rate of 36% in men and 21% in women [180]. Prospective studies on the prevalence of fractures and pain that is attributable to OP in SCD are desperately needed to inform treatment guidelines and interventions. 

The current management recommendation for OP in SCD is borrowed from the endocrine and thalassemia literature with an emphasis on primary prevention. Adequate vitamin D and calcium intake starting during skeletal development in childhood, encouraging weight-bearing exercise, early detection and treatment of hypogonadism or growth hormone deficiency, prevention and treatment of iron overload, are strategies that should be incorporated into routine SCD comprehensive care [157]. Prospective screening for OP annually (using dual energy X-ray absorptiometry (DEXA) of at least 2 separate sites) once patients reach age of anticipated peak bone mass is suggested to detect early deterioration in BMD [148, 181]. A detailed musculoskeletal evaluation should also be performed to rule out fractures of at-risk sites (hips, shoulders, and spine) particularly when vaso-occlusive pain becomes prolonged and poorly responsive to usual therapies as the occurrence of OP-related fractures may often go undetected. The use of bisphosphonates in secondary OP due to SCD has not been previously reported. Studies in thalassemia subjects suggest efficacy in reducing bone turnover with modest gains in BMD at lumbar spine but not femoral neck [157, 182]. A meta-analysis of seven clinical trials of bisphosphonates for secondary OP in children by Ward et al. concluded that the evidence was insufficient to support its use as standard therapy and supported the need for further evaluation [183]. In adults, however, bisphosphonates are widely used for secondary OP with acceptable efficacy and tolerability [184, 185]. There are currently no consensus guidelines for their use in SCD for treatment of secondary OP. Hormonal supplementation for hypogonadism may be effective in preventing and halting progression of OP in SCD; however concerns for increased risk of thrombosis (in females) and priapism (in males) will need to be addressed by further research [186–188]. Rigorous prospective studies of these agents in adults and children with SCD are needed to determine their short- and long-term safety, tolerability and efficacy in prevention and treatment of OP.

### 4.3. Myositis/Myonecrosis/Fascitis

Acute vaso-occlusion involving muscles, tendons, and fascia can occur in SCD [189]. It is seen in individuals with prior history of severe prolonged vaso-occlusive episodes and typically low hemoglobin F%. Anecdotal reports in adolescents suggest strenuous activity and muscle overexertion as seen with competitive dancing, video gaming, and other sports may contribute to the increasing incidence of sickle myositis and myonecrosis. The pattern of muscle involvement is usually symmetric and involves most often the proximal muscle groups [190]. Acute VOC involving muscles leads to myositis, myonecrosis, and eventual myofibrosis, which can result in long-term sequelae including contractures, indurations, and muscle atrophy. Patients usually present with acute focal pain, tenderness, and swelling of discrete muscles or groups of muscle/compartments that is out of proportion to their usual pain crisis and described as “different” from a typical sickle pain episode. Progression to involve the underlying fascia culminates in an acute compartment syndrome [191]. Superinfection with *Staphylococcus aureus, Streptococcus pneumoniae*, or other organisms may occur with spread to underlying bones and joints leading to progressive organ damage, sepsis, and multiorgan failure. Detection of an elevated LDH, CPK, (muscle fraction) and myoglobin in serum may be suggestive; however it is not present in all cases. Elevated CRP may suggest concurrent infection and warrants empiric antibiotic therapy. Confirmation is made by evidence of muscle inflammation and necrosis by MRI or on muscle biopsy showing inflammation, edema, and necrosis of muscle fibers with collagen deposition and fibrosis [190, 192].

Although myositis, myonecrosis, and fasciitis are difficult to distinguish clinically, initial treatment modalities are the same and include bed rest, short-term immobilization, intravenous fluid hydration, anti-inflammatory agents, and opioid analgesics for pain relief [193]. There is not much written in the literature on the optimal management of this complication in SCD; however in diabetic myonecrosis, exercise or physical therapy typically exacerbates pain and extends infarction, so should be avoided [193]. Very rarely with myofasciitis, an acute compartment syndrome may occur requiring surgical intervention to decompress the muscle compartment and/or excise any calcified necrotic material that would compromise organ perfusion. The novel use of low-level laser phototherapy is currently being investigated in animal models of ischemic muscle injury as a therapeutic modality to reduce myonecrosis by promoting muscle repair and angiogenesis targeting type I and III collagen fibres [194]. Other novel therapies are being evaluated for their benefit in sickle ischemia reperfusion injury such as is seen with myonecrosis and include anti-inflammatory agents such as sulphasalazine, an NF*κ*B inhibitor and IVIG [195]. Sulfasalazine is believed to reduce expression of VCAM, ICAM, and E selectin on endothelial cells in the microcirculation and attenuate the effect of reperfusion injury thereby improving microcirculatory blood flow in sickle mice, while IVIG is believed to reduce leukocyte adherence to endothelium and improve microcirculatory blood flow in transgenic sickle mice [196, 197]. Further research on the applicability of these interventions in humans is warranted.

### 4.4. Avascular Necrosis (AVN)

The complete disruption of vascular supply to the articular surfaces and ends of long bones, particularly the femoral and humeral head and spine, results in avascular necrosis or AVN [146, 198, 199]. Scientific advances in chronic bone disease have lagged behind the impressive improvements in survival for SCD, and AVN remains the leading cause of crippling disability in this population [146, 198, 200–204]. Approximately 50% of individuals with SCD will develop some form of bone fragility syndrome (osteonecrosis (AVN), compression spine fractures) by age 35 years. Consequently, AVN is a major cause of frequent hospitalizations, increased health care utilizationts, and poor quality of life in these patients [198, 205–208]. In adolescents and adults with SCD AVN typically involves the epiphyseal bones of the hip, shoulder, and spine; however any joint could be affected. Risk factors for AVN include recurrent vaso-occlusion (VOC), male gender, high hemoglobin, low hemoglobin F, vitamin D deficiency, and alpha thalassemia trait [162, 201, 207, 209–218].

Recent studies in SCD suggest a 26% prevalence of AVN among children with hemoglobin SS (mean age 9.8 years), and in adults it is 48.6% with a mean age of 26.7 years with a four-year progression rate of 67% [205, 212]. In a report by Ware et al., 41% of adults with hemoglobin SS over the age of 15 years had AVN of a bone [219]. Koduri et al. reported that silent AVN occurred in 41% of children aged 4–28 years with spine and shoulder involvement occurring in 27% and 28% cases, respectively. Fourteen to 23% of individuals with Hgb SC develop AVN [211]. In Jamaica, Lee et al. reported that the likelihood of developing AVN was 82% between the ages of 10 and 29 years [220]. Earlier reports on the prevalence of AVN in SCD were grossly underestimated particularly since 47% of patients with hip disease and 79% with shoulder disease had no symptoms at diagnosis [198, 215]. Bilateral hip involvement is seen in about 40–91% of SCD patients with femur AVN with a 3–5 year progression time to complete collapse (without intervention) particularly in older adolescents and adults who typically fail conservative management and develop a secondary degenerative arthritis [202, 217, 221–223]. Progression to complete joint collapse is usually rapid even in asymptomatic cases, and results of joint replacement are quite poor [202, 224, 225].

Early diagnosis of AVN is of critical importance as outcomes of conservative management are dependent on disease severity. A multidisciplinary approach preferably in a specialized center with expertise in SCD is needed to achieve optimal treatment outcomes and should involve orthopedics, hematology, physiotherapy, and nutritional expertise [146]. Treatment goals are primarily to relieve symptoms, prevent disease progression, and improve function of affected joints. Non-weight-bearing exercises that strengthen the affected limb girdle and improve range of motion are recommended. Presence of nutritional deficiencies that affect bone growth and development should be ruled out particularly in children such as vitamin D and zinc deficiency and protein energy malnutrition. Failure of conservative measures and/or progression to joint collapse, fractures, or debilitating pain necessitates surgical interventions such as core decompression, arthroscopy, arthroplasty, or total joint replacement (TJR). Perioperative management for orthopedic procedures in sickle cell patients should include attention to hydration and oxygenation, simple or exchange transfusions, and close monitoring for hypoxemia, development of vaso-occlusive crisis and acute chest syndrome [226–229].

The use of core decompression although established as an effective treatment for stage 1 AVN was showed to have no superiority over intensive physiotherapy in a randomized controlled trial (National Osteonecrosis Trial in Sickle Cell Anemia Study Group) [206, 230]. Novel therapies currently under investigation for early stage AVN include extracorporal shock wave therapy [231, 232]. Stem cell therapy is a promising new method in the management of stage 2 and 3 disease (with or without osteotomies) and utilizes vascularized bone grafts and/or autologous volume reduced bone marrow injected into femoral head following core decompression to promote healing and revascularization of affected bone and joint [233].

The treatment of stage 4 disease is more complex, and depending on size and location of the necrotic zone and the pathology of the adjacent bone, resurfacing or short-stem hip arthroplasty can be performed. Conventional total joint replacement (TJR) is still however the gold standard. This poses a challenge for the younger patient before the second decade of life who continues to have longitudinal bone growth in the unaffected limb and is more active resulting in length discrepancies, prosthesis instability, and muscle imbalance. There have been conflicting reports on the success of joint replacement surgery for severe AVN in SCD, and prosthesis failure is especially common in these patients. The typical lifespan of a replaced joint is 5–10 years with individuals suffering loosening of the prosthesis over time. Patients may continue to have continued pain and limitations of mobility even after arthroplasty. A recent review reported a revision rate of 31–63% with less than ten years of followup compared to 10% in nonsickle patients [201, 217, 234–236]. The longevity of a replaced hip has increased significantly with modern surgical techniques, modern implants that use materials such as ceramic and polyethylene, and improved disease management strategies with a concomitant reduction in complication rates [236].

The use of chronic hypertransfusion (CHT) and hydroxycarbamide (HU) to modulate disease severity and prevent sickle vaso-occlusive complications has not been shown to conclusively reduce the risk of AVN in SCD [237]. On the contrary, there are reports of new and worsening AVN among SCD patients on HU [238–240]. Prospective screening for AVN using standardized tools such as the Children's Hospital Oakland Hip Evaluation Scale (CHOHES) should be incorporated into comprehensive care for all individuals with SCD [241]. Larger prospective longitudinal studies are needed on the incidence, prevalence, risk factors, and progression rates of AVN with various interventions in the hydroxycarbamide era. Randomized controlled trials of improved joint and spine rehabilitation techniques in SCD would shed more light on optimal therapeutic options.

### 4.5. Leg Ulcers

Ulcerations of the skin and underlying tissues may occur in SCD most commonly involving the medial and lateral aspects of the ankle. Delayed healing leading to skin ulceration may also occur at postsurgical sites of after mild trauma. Risk factors associated with the development of leg ulcers in SCD include trauma, infection, severe anemia, high hemolytic rate (elevated LDH and reticulocyte count, low hemoglobin and hemoglobin F), geographic location, socioeconomic status, and venous incompetence [242–244]. Recent studies have proposed an association between hemolysis-induced vasculopathy and a hemolytic subphenotype in SCD that presents clinically with increased incidence of leg ulcers, pulmonary hypertension, and priapism [245]. Genetic factors such as HLA B3 and Cw4 are associated with a 17-time increase risk of leg ulcers in SCD [246]. Various candidate gene and genome wide association studies on leg ulcer susceptibility in SCD are ongoing [247].

The epidemiology of leg ulcers in SCD is not well defined as the clinical impact on patients is often underestimated by clinicians since it is not immediately life-threatening complications. Environmental factors and geography influence the prevalence of leg ulcers, and in the Unites States approximately 2.5% [242] of persons with SCD will present with a leg ulcer whereas in Africa the range is 1.5–13.5% and in Jamaica it is over 40% [246, 247]. Patients with leg ulcers are typically older in age, have homozygous SS disease, and present with large ulceration areas (median diameter of 12.4 cm) that have persisted for prolonged period of time (median duration of 29 months) [243]. Common sequelae include superinfection, ankle stiffness and edema, osteomyelitis, pathological fractures, severe pain, mood disorders, and poor-health-related quality of life [243].

The pathogenesis of chronic leg ulcers in SCD is poorly understood and quite complex. Factors that predispose to chronic ulceration in SCD include poor skin perfusion (due to mechanical obstruction to flow from vaso-occlusion), increased local edema from venous incompetence, abnormal autonomic vascular control (inadequate venoarterial response to leg lowering and secondary venous hypertension) [248], microvascular thrombosis, decreased oxygenation, reduced nitric oxide bioavailability (impaired endothelial function), and minor trauma [246, 249]. Histopathological sections of sickle leg ulcer biopsies show similarities to diabetes and vascular disease-related ulcers with intimal proliferation, neovascularization, and perivascular proliferation at the base of the ulcers suggesting a role for thrombosis. Raised edges with hyperpigmentation and hyperkeratosis are characteristic, and ulcers may penetrate deep into fascia or even involve periosteum of bone.

Primary prevention is the main focus of the management of leg ulcers in SCD. A detailed review of prevention strategies and the treatment of leg ulcers by Eckman provides practical management insights [246]. All patients with SCD should be educated on avoiding ill-fitting shoes and even minor trauma to extremities (such as insect bites, use of extremities for drawing blood or intravenous therapy) with prompt treatment if trauma does occur [246]. The use of support stockings with limb elevation for edema, emollients to prevent cracking and drying of skin, and the use of cotton socks over nylon or synthetic fibers are commonly used secondary prevention strategies.

Once an ulcer is established, treatment can be very frustrating for both patient and medical team. A multipronged approach that provides for gentle debridement (with DuoDerm hydrocolloid dressings, Unna boots), prevention and control of local edema, control of infection, provision of a local healing environment with topical agents, and systemic treatment of the disease and other identified micronutrient deficiencies has provided the best treatment outcomes. Zinc is a trace element that plays an important role in immune function and wound healing. Small but randomized studies have shown efficacy of oral zinc sulfate 220 mg three times a day in promoting rapid healing of leg ulcers [250]. This may be particularly useful in individuals with SCD who have high rates of zinc deficiency. Skin grafts and myocutaneous flaps have been used for recalcitrant ulcers resistant to local therapy; however results are equivocal as there remains a high risk of thrombotic occlusion of graft microvasculature [251]. Short-duration chronic hypertransfusion therapy may reduce anesthetic risk and improve chances of graft success along with anticoagulants and or antiplatelet agents [249, 252]. Topical antibiotics have been shown to improve healing time compared to controls; however the use of parenteral or oral antibiotics is reserved for ulcers complicated with systemic infection or osteomyelitis [253].

Chronic leg ulcers will recur between 25 and 50% of the time, and retreatment will be needed. There are several anecdotal [254] reports on the use of hyperbaric oxygen, arginine butyrate, topical herbal applications, and topical growth factors in the treatment of SCD leg ulcers without randomized trials to confirm their utility. Recent advances in the management of leg ulcers in diabetics include topical applications of analgesics, including opioids for pain [255], topical application of a platelet-derived growth factor prepared either by autologous (Procuren) or by recombinant technology (Regranex) [256], and the use of cultured skin grafts. The efficacy of these agents will need to be confirmed by randomized clinical trials in SCD.

### 4.6. Osteomyelitis/Septic Arthritis

Bacterial infections involving the cortical bone (osteomyelitis) and joint space (septic arthritis) have been commonly reported in SCD, particularly in association with avascular necrosis and bone infarcts [257, 258]. The prevalence of OM is lower in individuals with the Bantu haplotype, and it may occur as a complication of severe leg ulcers [259]. The most common etiologic organism in sickle OM is salmonella followed by *Staphylococcus aureus* and enteric gram-negative bacilli [260, 261]. The femur, tibia, and humerus are the most commonly affected sites. Making the diagnosis of OM on clinical grounds in SCD is extremely difficult as the signs and symptoms of OM mimic those of an acute sickle vaso-occlusive episode or bone infarct. Acute long bone infarcts are however fifty times more common than bacterial OM in SCD [262]. It is important to make the distinction between sickle vaso-occlusion and OM to prevent progressive bone and joint damage with treatment delays and to avoid unnecessary exposure to a prolonged course (six weeks) of antibiotic therapy. Pain, swelling, and fever may be associated with elevated white blood cell counts and inflammatory markers such as CRP [263]. The utility of blood, bone, and joint aspirate cultures is limited since most patients would have received antibiotics within the first twenty-four hours following a febrile episode. The presence of sterile white blood cell collections in bone and joint aspirates is enough to confirm diagnosis.

Early changes on plan radiography are nonspecific and include periosteal reactions, osteopenia, and lucent areas within bone. Ultrasonography is a rapid noninvasive way of showing extra osseous pathology associated with OM; however it has only 74% sensitivity, and findings are nonspecific [264, 265]. Computer tomography may show subperiosteal fluid collections with deeper collections correlating more with presence of infection [266, 267]. The use of radio-labeled isotopes and tagged leukocyte scans is less frequent as false negatives and positives occur frequently. Magnetic resonance imaging (MRI) with gadolinium enhancement increases the sensitivity of diagnosis; however there is still overlap between the changes seen with infection and infarction. The final diagnosis of OM is usually made using a combination of clinical, radiographic, and laboratory parameters coupled with a high index of suspicion. Sometimes surgical aspiration is needed to drain a localized fluid collection in subjects with poor response to antibiotics [146, 268, 269].

Septic arthritis (SA) is estimated to occur in 0.2 to 5.4% of individuals with SCD and usually occurs in an osteonecrotic joint and is caused by similar organisms to OM [270–272]. Septic arthritis is also a common complication following hip arthroplasty in SCD and a reason for joint failure. Concurrent involvement of multiple joints is not uncommon. The diagnosis of septic arthritis also requires a high index of suspicion. The presence of pain, swelling, and immobility around a joint is usually assumed to be from a typical vaso-occlusive episode. Persistence of symptoms of pain and swelling with or without fevers should prompt imaging studies and further laboratory workup. Serum CRP should be obtained and if elevated should raise suspicion for septic arthritis. The CRP is typically the first marker to be elevated and the first to respond to treatment. Delayed diagnosis is associated with rapid joint deterioration and collapse [146].

The mainstay of treatment for both OM and OA is a prolonged course of antibiotics. Initial use of parenteral antibiotics against salmonella, staph, and enteric gram-negative organisms is prudent to achieve rapid bactericidal blood levels. A six-week course of antibiotics is recommended for confirmed cases. Physical therapy to improve joint function and avoid muscle wasting should be encouraged once pain improves.

## 5. Neurological Complications

### 5.1. Ischemic Stroke

Data from the Cooperative Study of Sickle Cell Disease (CSSCD) revealed that stroke occurred in 11% of children with hemoglobin SS (HbSS) below the age of 20 years and 24% of adults by age 45 [273]. However, the use of transcranial Doppler ultrasonography (TCD) in the past two decades to identify persons at high risk for ischemic stroke and the prophylactic management of those patients with chronic transfusion has dramatically reduced the incidence of childhood stroke to approximately 2-3% [274–276]; this is discussed further below. Consistent with previous CSSCD findings, a recent retrospective study confirmed that high systolic blood pressure, leukocytosis, and severe anemia were correlated with MRI-documented brain injury in children with sickle cell anemia [277]. Seizure, sensory, and motor events were associated with the highest risk for brain injury, while the less specific problems of headache and poor school performance were not correlated with increased risk. Similarly, acute CNS events in children with sickle cell disease were associated with older age, history of stroke, transient ischemic attack, or seizure, neurologic symptoms, focal neurologic exam findings, and an elevated platelet count [278].

The acute management of overt stroke has not changed substantially in the past three decades. Patients presenting with clinical evidence of acute cerebral ischemia receive an immediate CT scan of the brain to rule out intracranial hemorrhage and are initially managed in an ICU where they receive simple or exchange transfusion to lower circulating HbS levels to ≤30% and correct anemia to a hemoglobin level of approximately 11 g/dL. Subsequent management requires chronic simple transfusion or erythrocytapheresis to maintain HbS at ≤30% (or a community standard level of ≤45% [279]). Of major clinical benefit has been the availability of the oral iron chelator deferasirox, which in the United States has almost completely replaced subcutaneous infusion of desferrioxamine as an effective means of controlling the burden of iron overload from chronic erythrocyte transfusion [280]. However, approximately 15–20% of patients who receive chronic transfusion for secondary stroke prevention will experience additional CVAs [281, 282]. A more recent study reported recurrent cerebral infarcts, both overt and silent, in 45% of children receiving chronic blood transfusion therapy for secondary stroke prevention [283]. Moreover, many adult patients and their providers choose to discontinue transfusion given its associated morbidities, including alloimmunization, iron overload, and infection [284, 285]. The multicenter randomized SWiTCH trial compared the administration of hydroxyurea plus phlebotomy with standard management (chronic transfusion plus iron chelation) for the prevention of secondary stroke and iron overload in patients who had experienced an initial CVA (clinicaltrials.gov #NCT00122980) [286]. However, this recently completed trial did not show equivalency of the “experimental” regimen with standard management.

In contrast, primary prevention of ischemic stroke using TCD screening and prophylactic chronic transfusion of persons at high risk for stroke indicated by abnormal velocities (>200 cm/sec) has dramatically lowered the overall incidence of stroke [274–276, 287]. In most large sickle cell centers more patients currently receive chronic transfusion for primary stroke prophylaxis than for secondary prevention. The STOP-II study determined that regular transfusions for primary stroke prevention could not be stopped safely, even in patients with a normal MRA whose TCD studies had normalized [288]. Within one year after stopping, about half the children had been restarted on transfusion, either because of stroke or stroke risk, or other indications such as recurrent pain or severe acute chest syndrome. While the necessary duration of transfusion remains to be determined, STOP II indicated ongoing risk in most cases even after prolonged transfusion, arguing for the need for continued treatment. Thus, an important question is the potential role of hydroxyurea in primary stroke prevention. Early findings from nonrandomized clinical series suggested that hydroxyurea might be an alternative to transfusion for primary stroke prevention because of its efficacy in decreasing TCD velocity [289–291] and reducing the overall occurrence of CNS events [287]. The recently initiated multicenter TCD With Transfusions Changing to Hydroxyurea (TWiTCH) trial (a phase III noninferiority study comparing regular transfusion to hydroxyurea in children with abnormal transcranial Doppler studies to prevent stroke) involves the randomization to hydroxyurea treatment or continuation of standard transfusion to determine the efficacy of hydroxyurea in primary stroke prevention.

### 5.2. Silent Cerebral Infarction

Silent infarcts occur in approximately 20% of children with sickle cell anemia and are associated with significant compromise of neurocognitive performance as well as an increased risk for subsequent overt stroke [292, 293]. Children with SCA and MRI confirmed that silent infarcts have lower intelligence quotients (IQs) than those with a normal MRI [294]. The international multicenter randomized silent infarct/transfusion (SIT) trial is addressing the utility of chronic transfusion compared with standard observation in preventing the deleterious effects of silent infarcts on the CNS (clinicaltrials.gov# NCT00072761). Recent data indicate that silent cerebral infarcts occur despite regular blood transfusion for secondary stroke prevention in children with sickle cell anemia [283]. However, there is suggestive evidence that hydroxyurea may prevent or reduce the progression of silent cerebral infarcts [295]. In general hematopoietic stem cell transplant has been effective in preventing reoccurrence of CNS events [296], although progressive brain parenchymal damage has been reported following transplant [297].

### 5.3. Neurocognitive Impairment

Although neurocognitive deficits are usually associated with overt stroke in sickle cell disease, compromise is almost as significant in persons with silent infarcts and also occurs in those who have no apparent lesions on cerebral MRI [294, 298, 299]. Evidence of impairment occurs in the areas of attention, executive function, visio-motor function, verbal performance, and memory [299, 300]. Adults with HbSS were recently found to have lower performance IQ scores, associated with the severity of their anemia and increasing age [301]. In this study 138 adults with sickle cell anemia and 37 controls were evaluated with a battery of cognitive tests; neurocognitive dysfunction, undetected brain injury, or both affected most of the sickle cell subjects. Areas of executive functioning, reading, and mathematical ability were particularly affected. Sixty-three percent of sickle cell subjects had neuropsychological dysfunction or abnormal findings on MRI, 38% had neuroimaging abnormalities, including silent infarcts and hippocampal atrophy, and 32% scored below 86 on the Wechsler Adult Intelligence Scale (WAIS) IQ scale. In contrast, 15% of the normal adult population scored below 86 on the WAIS scale. After controlling for age, gender, and education, sickle cell patients performed significantly worse than controls on the WAIS Processing Speed Index score and the Woodcock-Johnson score, tests of reading, math, and ability to follow directions. In addition, subtest scores of the Test of Everyday Attention, which measures flexibility of thought and attention, were significantly decreased in sickle cell patients, particularly related to age. Volumetric MRI measurements showed no significant differences in total gray matter or volume of the hippocampus between patients and controls, but there was a nonsignificant reduction in brain volume with older age among adults with sickle cell anemia. Although lacunae were more frequent in these patients, the lesions were not related to neurocognitive function. The results of this study suggested that standardized and comprehensive neuropsychological assessment may be valuable in the management of adult patients with sickle cell disease. Recently, diffusion-weighted imaging (DWI) MRI data have been analyzed retrospectively to quantitate changes in tissue integrity that are present in normal-appearing white matter [302]. Subtle changes in white matter structure were related to processing speed, suggesting that modifications in tissue integrity may be used to predict cognitive morbidity of sickle cell patients.

Cerebral blood flow (CBF) has also been shown to influence neurocognitive function. Children with sickle cell anemia and abnormal CBF velocity, measured by TCD, perform more poorly on tests of verbal intelligence and executive function [303]. Cerebral blood flow velocity has been related to language functioning in children with SCD, particularly in the domain involving syntactical ability [304]. The results of this study underscore the need for neuropsychological assessment when abnormal flow velocities are detected by screening TCD. Examination of the relationship between CBF and neurocognitive function in children with sickle cell anemia (and a normal TCD) showed that CBF inversely correlated with both full-scale and performance IQ [305]. The authors concluded that assessment of increased CBF by continuous arterial spin-labeling MRI may allow interventions to modify the risk of neurocognitive impairment from sickle cell anemia, potentially before silent infarcts or abnormal CBF velocity develop.

No treatment has been demonstrated to improve neurocognitive dysfunction in this population, but results of a multicenter controlled randomized trial of short-term (6-month) transfusion intervention are pending (clinicaltrials.gov#NCT00850018). In addition, there is suggestive evidence from a report of 15 children treated with hydroxyurea, who scored higher on tests of verbal comprehension, fluid reasoning, and general cognitive ability than children not on the drug [306]. Benefit might ensue from lessened anemia, improving oxygen supply to the brain, and reduced fatigue due to illness. More definitive evidence of the effects of chronic transfusion and hydroxyurea on neurocognitive performance is expected from the SIT trial and long-term followup of the BABY HUG population. Interventions targeting specific areas of neurocognitive dysfunction (such as memory) have had some success but have been very limited in scope [307].

### 5.4. Moya-Moya

Moya-moya syndrome has been increasingly recognized following stroke in children with sickle cell anemia and has been associated with intellectual decline, particularly in performance IQ [308], as well as a markedly increased risk for subsequent cerebrovascular event (stroke or transient ischemic attack (TIA)) [309]. Management of this complication has ranged from continuation of chronic transfusion (for secondary stroke prevention) to a variety of neurosurgical direct and indirect revascularization techniques. Two recent series have been reported, each involving 12 patients with sickle cell anemia, previous stroke and/or TIA, and the presence of moya-moya syndrome despite chronic transfusion management [310, 311]. The total of 24 patients underwent unilateral or bilateral encephaloduroarteriosynangiosis (EDAS)/pial synangiosis at an average age of 11-12 years and had a mean followup of approximately four years. Except for two subjects who had relatively limited CVAs within three weeks of neurosurgery, the patients remained stroke-free during their followup and were neurologically stable or improved, indicating that this intervention should be strongly considered for this very high risk subpopulation.

## 6. Ophthalmologic Complications

Vaso-occlusion can affect any vascular bed in the eye, including the conjunctiva, anterior segment, retina, choroid, or optic nerve with potentially blinding consequences [312]. Little is known about the role of genetic and environmental modifiers in the ocular manifestations of the sickle cell disease (SCD).

### 6.1. Orbital Involvement

Although rare, orbital compression syndrome (OCS) has been reported as an ocular manifestation of sickle cell disease [313–315]. In a series of 3 cases, sphenoid bone infarction led to a subperiosteal hematoma and an inflammatory response that resulted in acute proptosis, periorbital pain, restricted motility, and compressive optic neuropathy, known as the orbital compression syndrome (OCS) [316]. OCS is often diagnosed by magnetic resonance imaging (MRI), and most cases resolve with medical management without the need for surgical intervention. Timely treatment with corticosteroids may be helpful in relieving the orbital pressure caused by the inflammation and reversing the associated optic neuropathy. Antibiotic coverage is also recommended if a coexisting infection is suspected or cannot be excluded. In cases with failed response to medical management, surgical drainage of the subperiosteal collection is indicated urgently [313–315].

### 6.2. Conjunctiva

One of the early vaso-occlusive features in SCD may be seen in the conjunctival vasculature. Abnormalities take the form of transient saccular and sausage-like dilations packed with red cells, resulting in dark red, comma-shaped vessel segments, most notably in the inferior bulbar conjunctiva [317]. Paton reported that these comma signs were more common in SS than in SC disease and were uncommon in patients with high HbF levels. The results of a study investigating the influence of clinical, laboratory, and genetic features on conjunctival and retinal vessel alterations indicated that low levels of Hb and hematocrit may be risk factors for conjunctival alterations. Conjunctival abnormalities were more evident in patients with SS disease [318].

Conjunctival blood flow improves following transfusion. The comma signs are also noted to diminish under heat from slit lamp beam illumination, which probably induces vasodilation. Abnormalities increase with pharmacologic agents inducing vasoconstriction, and these effects are reported to be more apparent in the pediatric patient group [319, 320]. Histopathologic examination of these conjunctival vessels demonstrated endothelial proliferation, aggregation of red blood cells in the distal portion of capillaries, and dilatation and thinning of the proximal segments of the vessels [321]. Visual acuity is not affected. The “comma sign” vessels in conjunctiva are an excellent diagnostic tool for sickle cell disease, but they are absent in sickle cell trait.

### 6.3. Anterior Chamber

The anterior chamber of the eye, normally filled with aqueous humor, has low oxygen tension, low pH, and high ascorbate concentration (a reducing agent) and may be filled with red blood cells following trauma or surgery, resulting in a condition called hyphema. When erythrocytes and leukocytes consume oxygen and liberate CO_2_ and lactic acid leading to acidosis, even sickle trait red cells become sickled. They then become trapped, since deformed and less pliable sickle cells are unable to pass through the 0.3–2 *μ*m pores of the aqueous outflow apparatus, namely, the trabecular meshwork and intraocular pressure increases [322, 323]. Only a moderate increase in intraocular pressure may cause a reduction in perfusion of the optic nerve head and retina, and an increased risk for optic atrophy and retinal artery occlusion, resulting in irreversible vision loss [324]. The presence of blood in the anterior chamber should be considered an emergency and should prompt an eye examination, including intraocular pressure measurement. Intraocular pressure should be around 25 mmHg or less [325, 326]. Anterior chamber paracentesis and intracameral injection of tissue plasminogen activator (t-PA) were reported to successfully normalize pressure and the vision in a child with posttraumatic hyphema, thrombosis in the trabecular meshwork and consecutive acute, secondary glaucoma [327]. Paracentesis by itself is often sufficient. Transcorneal oxygen therapy may also reduce the intraocular pressure in patients with glaucoma induced by sickle cell hyphema [328], by converting rigid erythrocytes to pliable ones, which can then escape from the eye through the trabecular meshwork.

### 6.4. Posterior Segment Manifestations

Posterior segment findings include retinal hemorrhages and exudates, angioid streaks, chorioretinal infarctions, vitreous hemorrhage, central or branch retinal artery occlusion, and proliferative sickle cell retinopathy (PSR).

#### 6.4.1. Salmon Patch Hemorrhage

Salmon patch hemorrhage represents a well-defined area of hemorrhage located within the superficial retina, between the sensory retina and its internal limiting membrane. It usually occurs in the midperipheral retina adjacent to an intermediate-sized arteriole [312]. Although the hemorrhage is initially bright red, it may turn salmon-colored over time because of progressive hemolysis, and resorption may result in a retinoschisis cavity. If the retinoschisis cavity contains refractile, copper-colored granules, it is called an iridescent spot. Histologically, these deposits contain hemosiderin-laden macrophages [329, 330].

#### 6.4.2. Black Sunburst

**Figure 1:**
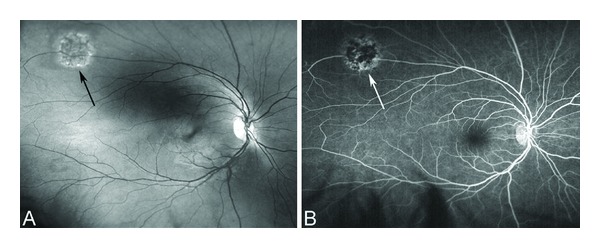
(A) Ultrawide field fundus photograph of the right eye shows a black sunburst lesion (arrow), a flat, round, black patch along the superior arcade temporally. (B) Ultrawide field fluorescein angiogram of the right eye in the arteriovenous phase demonstrates the staining of the sunburst lesion (arrow).

Intraretinal hemorrhages may track into the subretinal space, dissecting between the neurosensory retina and the retinal pigment epithelium (RPE), resulting in RPE migration into the site and forming the stellate and spiculate hyperpigmented lesion known as a black sunburst. The black sunburst lesion appears as a flat, round-to-oval, black patch about 0.5–2 mm in size (Figures 1(a) and 1(b)). Glistening refractile granules, similar to those in iridescent spots, may be present [312]. A large amount of intraretinal and subretinal hemorrhage may rarely alter the extracellular matrix or fibrous component of Bruch's membrane, allowing development of spontaneous choroidal neovascularization growing within the black sunburst lesion [331].

#### 6.4.3. Vasoocclusions

**Figure 2:**
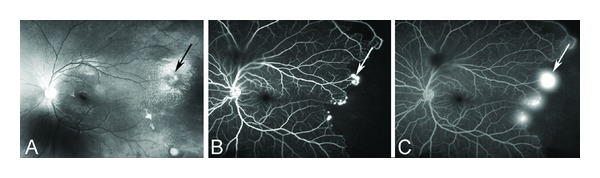
(A) Fundus photograph of new vascular formations characteristic of sickle cell disease called sea fan formations (arrow), which tend to occur in the temporal periphery. (B) Fluorescein angiogram in the arteriovenous phase shows peripheral nonperfusion and sea fan neovascularization at the border of vascularized and nonvascularized retina (arrow indicates the largest sea fan). (C) Late phase of the angiogram shows fluorescein leakage from sea fans as the dye study progresses.

**Figure 3:**
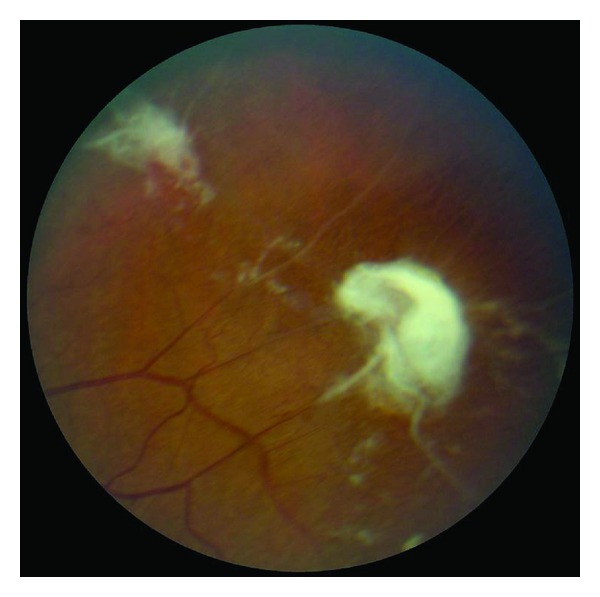
Color fundus photo shows a white autoinfarcted sea fan in the midperipheral retina with white, fibrous tissue remnants adherent to the cortical vitreous (top). Also note white, occluded retinal vessels.

Vaso-occlusions may occur in the retina or less commonly in the choroid of patients with sickle cell disease. In the retina, the initial vaso-occlusions typically occur in the peripheral retinal vasculature and mainly involve capillaries and precapillary arterioles. In more advanced retinopathy, occlusions can occur in any vessel within the peripheral retinal vasculature and can result in new vascular formations characteristic of sickle cell disease, such as sea fan neovascularization and hairpin loops [332] (Figures 2(a)–2(c), and 5(d)). Occlusion of major branch retinal arteries and even the central retinal artery may occur. One study reported that arteriovenous crossings were common sites of sea fan formation [333]. This same study demonstrated that these neovascular formations were very complex in that they can have multiple feeding arterioles from the retina and multiple draining venules [333]. Sea fans often autoinfarct, leaving white, fibrous tissue remnants adherent to the cortical vitreous (Figure 3).

Several case reports suggest that vascular occlusions also occur in the choroid [334, 335]. Choroidal nonperfusion is thought to result from occlusive events in the posterior ciliary arterial circulation. It was hypothesized that these occlusions may also be the stimulus for formation of choroidal neovascularization (CNV) and that this neovascularization may be involved in formation of some pigmented lesions, including some black sunburst lesions [332].

#### 6.4.4. Angioid Streaks

Angioid streaks are small breaks in Bruch's membrane, which separates retina from choroid. Angioid streaks resemble blood vessels, thus their name, and typically emanate radially from the optic nerve. These streaks are thought to be due to calcification and fragility of Bruch's membrane [312]. They are particularly common in the SS genotype.

#### 6.4.5. Macular Findings

**Figure 4:**
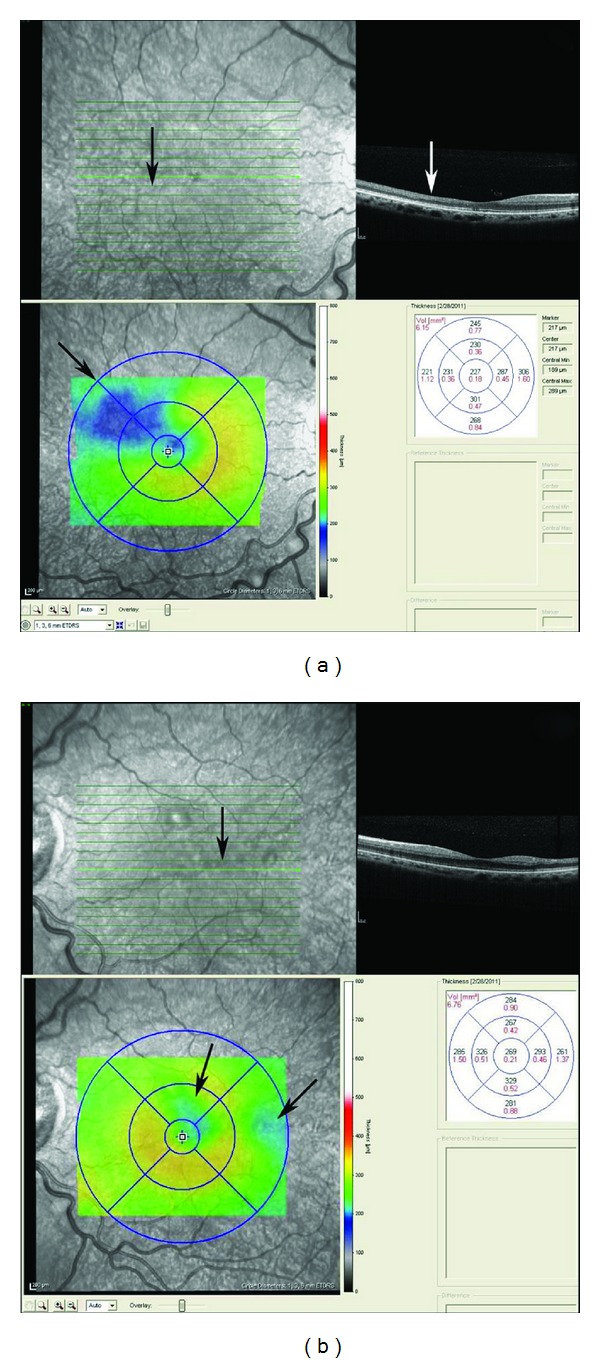
(a) Optical coherence tomography image of the right eye with a cross-section of the macula that demonstrates thinning of the inner layers (arrows), in the temporal aspect of the macula, whereas the nasal side of the macula (right) is normal in thickness. Thickness map below demonstrates the area of thinning, outlined by the blue color and shown by the arrow. (b) Optical coherence tomography image of the left eye shows a cross-section of the macula that demonstrates thinning of the inner layers, as indicated by the arrows, in the temporal aspect of the macula. Thickness map below demonstrates the two areas of thinning outlined by the blue color and shown by the arrows.

The macular findings in patients with sickle cell disease include abnormal perfusion and an enlarged foveal avascular zone (FAZ) due to vaso-occlusive episodes. Epiretinal membranes, schisis, holes, and neovascularization may also occur. The “retinal depression sign” was first described by Goldbaum as an abnormality in the reflection from the internal limiting membrane due to depression of the inner surface resulting from a small retinal infarct and subsequent atrophy [336]. These changes can now be demonstrated using noninvasive imaging techniques, such as optical coherence tomography (OCT), which offers high-resolution, cross-sectional images that correlate with the histological features in patients with retinal diseases. OCT enables visualization of tissue structures up to 2 mm below the surface [337]. The imaging is analogous to B-mode ultrasonography except that light instead of sound is used in this technique. Reversal of precapillary occlusions in the perimacular region may occur spontaneously; however, loss of the inner layers of the retina results in the retinal depression sign. Thinning of the temporal inner macula, presumably caused by ischemia and eventual atrophy of retinal ganglion cells and the nerve fiber layer, has also been shown with OCT [338, 339] (Figures 4(a) and 4(b)).

The presence of multifocal perifoveal occlusions was suggested to result possibly from a central retinal artery occlusion with migration of microemboli downstream in a 9-year-old boy with SS hemoglobinopathy [340]. In the presence of macular occlusive changes, emergency methods, such as anterior chamber paracentesis to lower the intraocular pressure, may be indicated in an attempt to prevent infarction and irreversible vision loss [341]. Additionally, immediate or regular exchange erythrocyte transfusions are sometimes used to improve visual outcomes or to prevent vision loss in the fellow eye. Evidence for efficacy is presently lacking.

Fluorescein angiography and automated perimetry of the central 30 degrees are more sensitive tests for detection of ischemic macular disease than visual acuity, and macular ischemia can be quantified by these two techniques [342]. Since many of the macular changes seen in sickle cell disease are subtle and can be missed by ophthalmoscopy, thorough evaluation, including angiography, OCT, and perimetry, is often important in the diagnosis and management of these patients.

Although infrequent, macular holes can also be seen in association with proliferative sickle cell retinopathy. The occurrence of fibroglial proliferation around the disc, with subsequent nasal traction on the macula, along with temporal traction resulting from vasoproliferative tissues near the temporal equator, has been suggested to play an important role in the formation of macular holes by stretching of the retina. The occlusion of perifoveal capillaries may be another possible mechanism, because it causes retinal atrophy, thinning, and possible hole formation [343].

#### 6.4.6. Retinoschisis

Retinoschisis is a rare complication of sickle cell retinopathy that is related to chronic low-grade ischemia of the inner nuclear layer [329]. It is characterized by a concave retinal elevation, inner-layer breaks, and a split pattern on OCT. Additional breaks in outer layers may complicate the course of the disease and result in retinal detachment in such cases [344]. Timely laser treatment may reduce the rate of full thickness retinal detachment in these patients.

#### 6.4.7. Disc Sign

The disc sign in sickling hemoglobinopathies is the appearance of isolated comma or hyphen-shaped small vascular segments on the surface of the optic nerve head. It is most likely due to piling up and subsequent deoxygenation of sickled erythrocytes caught at bifurcations in the capillary bed or precapillary arterioles and is most commonly seen in patients with HbSS disease. These transient plugs do not seem to result in any visual changes or loss [345]. They are analogous to, and resemble, the conjunctival comma sign.

#### 6.4.8. Proliferative Sickle Retinopathy (PSR)

**Figure 5:**
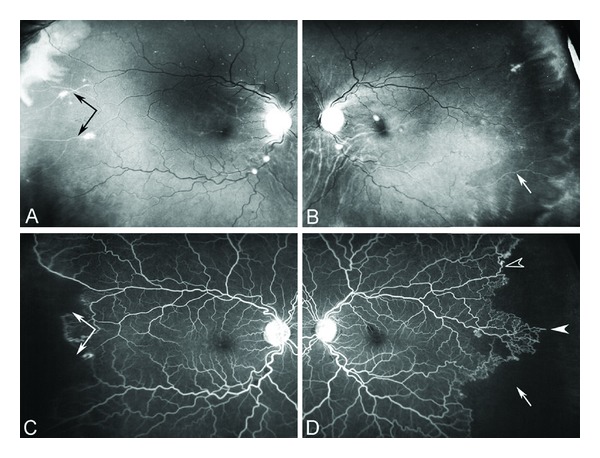
(A and B) Color fundus photographs of both eyes show white, occluded vessels (arrows) in the periphery bilaterally. (C and D) Ultrawide field fluorescein angiograms of the right and the left eye demonstrate the initial vascular remodeling at the junction of the perfused central, and nonperfused peripheral retina (arrows) includes arteriovenous (AV) anastomoses (black arrow head) and hairpin loops (solid white arrow head).

Arteriolar occlusion and loss of capillary perfusion in the peripheral retina are the most striking features of sickle cell retinopathy. They are generally more prominent in the temporal peripheral retina, especially superotemporally. The ischemic areas caused by these occlusions release substances that can stimulate angiogenesis [346]. The initial vascular remodeling at the junction between the perfused central and nonperfused peripheral retina includes the creation of arteriovenous (AV) anastomoses and hairpin loops [347] (Figures 5(a)–5(d)). Proliferative retinopathy occurs most frequently in HbSC disease with an incidence of approximately 33%. The condition is much less common in patients with S*β*-thalassemia; about 14% of patients with this genotype have PSR [346]. PSR can also be seen in patients with HbSS disease, though less commonly [348]. The peak prevalence of PSR in the HbSC genotype occurs earlier than in the SS type (about 15–24 years in men and 20–39 years in women) [349].

Goldberg and colleagues developed a widely accepted grading system detailing the stages of proliferative sickle retinopathy (Table 2) [320, 350].

Recent studies investigating the pathogenesis of retinal angiogenesis have focused on the balance between pigment epithelium-derived factor (PEDF), an anti-angiogenic factor, and vascular endothelial growth factor (VEGF), an angiogenic factor [351]. PEDF is a multifunctional factor that has both neurotrophic and antiangiogenic effects. In retinal ischemia, the development and progression of neovascularization may be influenced by the change in balance between VEGF and PEDF. It has been shown that the abnormalities in VEGF and PEDF levels are minimal before proliferative changes occur. The balance between PEDF and VEGF changes as the disease progresses to the proliferative stage. The main changes that have been observed include a decrease of the PEDF/VEGF ratio in the feeder vessels and active sea fan blood vessels, whereas in regression of neovascularization, there was an increase in the PEDF/VEGF ratio [352].

### 6.5. Treatment

#### 6.5.1. Indications

Observations in a Jamaican cohort study showed that spontaneous regression occurred in 32% of eyes with PSR. Permanent vision loss was rare among the subjects followed for 20 years until the age of 26 years [353]. Given the high rate of spontaneous regression (Figure 3) and the lack of progression of sea fans in some eyes, indications for treatment of retinal neovascularization have varied among different authors. Therapeutic intervention is usually undertaken in cases of bilateral proliferative disease, spontaneous hemorrhage, large elevated sea fans, or rapid growth of the neovascular tissue or in cases in which the fellow eye has already been lost due to PSR [354]. If peripheral neovascularization exceeds 60^°  ^of the circumference, therapy is usually implemented.

The goal is to induce regression of stage III lesions prior to a substantial hemorrhage and/or retinal detachment. With an early, successful treatment, the need for pars plana vitrectomy and/or scleral buckling, and their associated potential surgical complications, can be avoided [355–358]. Various techniques, including laser photocoagulation, cryotherapy and diathermy, have all been used to achieve involution of neovascular lesions. Specific methods of laser application include feeder vessel coagulation, local scatter coagulation with or without focal treatment of the sea fan, and 360-degree peripheral scatter delivery.

#### 6.5.2. Laser Photocoagulation

**Feeder Vessel Photocoagulation**

Prior to demonstration of the indirect effect of peripheral retinal destructive therapy on proliferating capillaries by suppression of vascular growth factors, laser treatment was used to occlude feeding vessels. Direct, heavy laser treatment was applied to the feeding arterioles and then to the draining venules. The sea fans were closed in 88% of eyes studied in a controlled clinical trial [359]. Although both xenon arc and argon lasers have been effective in achieving sea fan closure, the argon laser is currently more commonly used, because the risk of damage to Bruch's membrane is higher with the xenon arc [312]. Short-term complications included retinal or choroidal hemorrhage, peripheral choroidal ischemia, macular traction, and retinal detachment. Given the relatively high-power settings required to close arteries and arterioles (and the risk that such high-power laser light may damage Bruch's membrane), significant complications, including choroidal and choriovitreal neovascularization, have been observed. In a series of 53 eyes with PSR, at least 21 (39.6%) of the treated eyes developed chorioretinal or choriovitreal neovascularization [360]. Feeder vessel treatment is currently used only in refractory cases with recurrent bleeding and if scatter photocoagulation has failed.

**Scatter Photocoagulation**

The preferred therapy by many clinicians is either localized or widespread scatter photocoagulation because of its low rate of complications. It is performed similar to its use in proliferative diabetic retinopathy. The goal is to destroy nonperfused retina that is responsible for production of the angiogenic factors initially triggered by ischemia [361]. The laser scar enhances chorioretinal adhesion, potentially preventing or minimizing the extent of subsequent retinal detachment.

**Figure 6:**
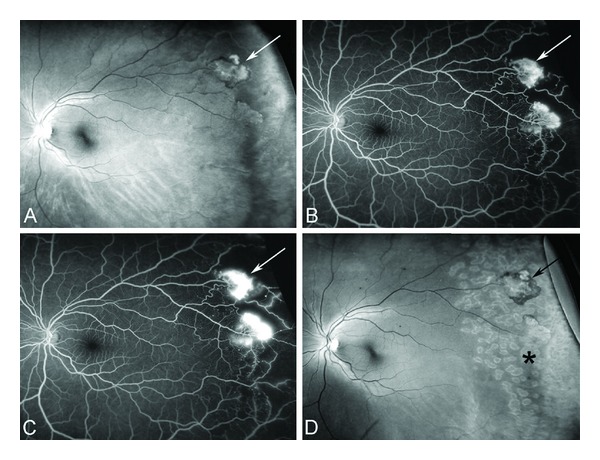
(A) Ultrawide field photograph of the left eye shows the sea fans with overlying preretinal hemorrhage in the superotemporal periphery as shown by the arrow. (B and C) Fluorescein angiogram delineates the nonperfused peripheral retina in the arteriovenous phase with 2 sea fans that leak as the study progresses. (D) Ultrawide field fundus image of the left eye demonstrates the guided scatter laser photocoagulation (asterisk) applied to the areas of nonperfusion noted on the fluorescein angiogram.

The new Optos P200MA Ophthalmic Imaging System may offer a distinct advantage over the traditional angiographic imaging in evaluating patients with sickle cell disease. The technology provides ultrawide field, high-resolution angiography, red-free, and full-color imaging, captured via a noncontact and nonmontage acquisition process, with up to a 200° internal view of the retina at one time. The images in Figures 1, 2, 5, and 6 were acquired with the Optos P200MA. Visualization of the periphery and peripheral lesions has contributed to the management and monitoring of these patients by allowing physicians to assess the status and progression of the disease beyond the posterior pole [362].

The P200MA uses low-powered laser wavelengths that scan simultaneously. While the red (633 nm) and green (532 nm) lasers are used to image the retina and the choroid, the blue (488 nm) laser permits the addition of fluorescein angiography with the ability to image almost the entire retina. Photocoagulation is now guided by findings on the wide-field angiography and is applied to areas of nonperfusion noted in these images adjacent to sea fans (Figures 6(a)–6(d)). The sea fans themselves are not focally or directly treated unless they are completely flat and not elevated into the vitreous chamber.

There are two retinal ablation techniques suggested for patients with PSR: sectoral and circumferential. Sectoral ablation is scatter photocoagulation only in the area around neovascularization. It has shown a reduction in vitreous hemorrhage and visual loss, without numerous complications [363]. However, in patients with vitreous traction in association with sea fan neovascularization, argon laser photocoagulation may sometimes result in retinal breaks and retinal detachment [364].

Regular followup is critical to assess progression of neovascular disease or treatment response following local, sectoral scatter photocoagulation. Despite scatter treatment, new neovascular fronds may develop in up to 34% of eyes [363].

A 360-degree peripheral scatter photocoagulation may be considered for an unreliable patient. In one series, peripheral circumferential retinal scatter photocoagulation to the peripheral zones of retinal capillary nonperfusion was shown to result in complete regression of 33% of preexisting sea fans [365]. As yet, there is no definitive evidence proving that the results of this approach are superior to the natural course of untreated disease or to sector scatter photocoagulation around individual sea fans [357, 366].

#### 6.5.3. Transscleral Diode Laser Photocoagulation

This technique was shown to be effective and safe in a case of PSR with vitreous hemorrhage and can be considered an alternative method in eyes when transpupillary laser application is not possible due to clouding of the media [367].

#### 6.5.4. Cryotherapy

For successful photocoagulation, the media must be sufficiently clear overlying the vasoproliferative tissue to allow the photocoagulation beam to form a sharp image on the retina. Vitreous hemorrhage or lenticular opacities can interfere with an adequate view of the retina and prevent successful photocoagulation. In such cases, the single freeze-thaw technique has been successful in approximately 70% of treated sea fans [368]. The vitreous traction and preexistent retinal thinning associated with PSR make the triple freeze-thaw cryopexy technique, however highly risky, because it may result in retinal breaks and detachment [369].

#### 6.5.5. Laser Vitreolysis

The neodymium: yttrium-aluminum-garnet (Nd : YAG) laser has been used in patients with PSR to reduce vitreoretinal traction by lysing localized, taut vitreous bands or to clear the media in front of the macula [370]. Safety is maximized when the bands are avascular or only minimally perfused and are located at least 3 mm from the retinal surface. Complications of posterior segment Nd-YAG laser treatment have been previously described and include choroidal hemorrhage, damage to the retinal pigment epithelium, and bleeding from perfused vascular bands [371–373].

#### 6.5.6. Pars Plana Vitrectomy and Scleral Buckling

PSR may cause vitreous hemorrhage that occasionally is severe or persistent and that requires surgical intervention. Patients with new vitreous hemorrhages may be followed for at least 6 months to allow spontaneous clearing of the media when there is no associated retinal detachment [354, 356]. Early surgical intervention is indicated if the hemorrhage is associated with rhegmatogenous retinal detachment.

Full-thickness macular hole is another indication for vitrectomy surgery in patients with PSR. The surgical approach usually includes vitrectomy and removal of epiretinal membranes [374, 375].

Vitrectomy and scleral buckling surgery carry significantly higher risks of intraoperative and postoperative complications in patients with sickle hemoglobinopathies. There has been, however, considerable advancement of vitreoretinal surgical techniques and instrumentation in the course of recent years, making this type of surgery much safer than ever before. The introduction of endolaser photocoagulation, intraocular diathermy, endoillumination, and, most recently, small gauge, microincisional surgery is one of the advances that has improved the rate of surgical success in patients with sickle cell disease.

Vitrectomy is often combined with scleral buckling in patients with enlarged or progressive tractional detachments or with a notable tractional component in association with proliferative disease. The success rate, however, for visual acuity improvement in a series of eyes with tractional retinal detachment with or without a rhegmatogenous component, requiring vitrectomy plus a scleral buckling procedure, was only 50% [356]. Complications associated with vitreoretinal surgery include iatrogenic retinal breaks, intraocular bleeding, secondary glaucoma, and anterior segment ischemia. In one series, the high incidence of iatrogenic tears and bleeding associated with the delamination technique leads to a switch in methodology to a segmentation technique that was performed only if necessary to remove the vitreal attachments. Scatter laser or feeder vessel treatment as an attempt to try to induce regression of preretinal neovascularization was found unnecessary. The authors also suggested that the vitreous hemorrhage may be allowed to clear spontaneously without detriment. Tractional retinal detachment was also observed without apparent progression in some patients [376].

Anterior segment ischemia was reported to occur in 71% of patients undergoing a scleral buckling procedure [377]. It was also reported to occur after pars plana vitrectomy with panretinal photocoagulation without scleral buckling [378]. The precipitating factors include significant rise in intraocular pressure, or other causes of reduced ciliary blood flow, caused by sickled erythrocytes.

### 6.6. Future Directions

Recent studies showed that the balance of PEDF and VEGF changes as the disease progresses to the proliferative stage [352]. This was due to an elevation of VEGF levels. Bevacizumab (Avastin) is a recombinant humanized monoclonal IgG1 antibody that binds to and inhibits the biologic activity of human VEGF in *in vitro *and *in vivo* assay systems. It is approved for the systemic treatment of metastatic colorectal cancer. However, studies reporting off-label use of intravitreal bevacizumab in patients with neovascular age-related macular degeneration have shown promising results. It has also been shown that the intravitreal injection of bevacizumab resulted in regression of retinal neovascularization in patients with sickle cell disease in an anecdotal report [379]. Controlled, clinical trials are required to assess the role of anti-VEGF agents in treatment of patients with PSR via the intravitreal route.

Although it has been assumed that sickled erythrocytes solely contribute to the vaso-occlusive processes seen in sickle cell disease, there is evidence that neutrophils may also contribute to the process [380]. The adhesion molecules responsible for PMN rolling and firm adherence, P-selectin and ICAM-1, have been shown to be elevated in the sickle cell retina and in sea fan formations. Cytokines, such as tumor necrosis factor (TNF)*α* and IL-1*α*, upregulate leukocyte adhesion molecule expression by vascular endothelial cells [381]. Both of these cytokines are elevated in steady-state sickle cell subjects, probably due to a low-grade inflammation caused by abnormal adhesion of sickled RBCs to endothelial cells [382]. Several reports have also demonstrated that some reticulocytes from sickle cell patients have the integrin *α*4*β*1 (VLA-4) on their surface [383, 384]. The adherence of reticulocytes via VLA-4, after administration of TNF-*α*, was demonstrated in a rat model of sickle-cell-mediated retinal vascular occlusion. Blocking VLA-4 with a cyclic peptide or monoclonal antibody has been shown to prevent sickled RBC retention in the retina in this model [385]. The mechanisms for retention of SS RBCs in retina and choroid appear identical. TNF-*α* stimulated retention of the SS red cells in choroid appears to be mediated by VLA-4, and this retention was inhibited by a VLA-4 antagonist (TBC772), a cyclic peptide, or by blocking one of the VLA-4's ligands, the CS-1 portion of fibronectin [386].

Recurrent vaso-occlusion and tissue infarction cause a repetitive inflammatory reaction in patients with sickle cell disease (SCD). The importance of inflammation is highlighted by observations that anti-inflammatory agents ameliorate SCD in humans and in animal models [195, 387]. Omega-3 polyunsaturated fatty acids (docosahexaenoic (DHA) and eicosapentaenoic (EPA) n-3 fatty acids) are precursors of anti-inflammatory and antiaggregatory eicosanoids. It was shown that the red cell and platelet membrane DHA and total n-3 fatty acids were significantly lower in patients with sickle cell disease complications. The data suggested that the reduced levels of DHA and EPA cause a greater tendency for inflammation and organ damage in patients with SCD [388, 389]. These findings provide insight into the biological basis of the potential benefits of n-3 fatty acid therapy in SCD. In a prospective, placebo-controlled, pilot clinical trial, the mean number of vaso-occlusive crises in five SCD patients decreased from 7.8 to 3.8 per year following oral intake of 0.25 g/kg/day of menhaden fish oil containing 12% EPA and 18% DHA (*P* < 0.01) compared with pre- and posttreatment crisis rates of 7.6 and 7.1 per year in five others taking the same doses of olive oil as placebo (*P* > 0.4) [390].

Recent studies have, therefore, shed light onto the pathogenesis of SCD and may permit identification of new treatment modalities in these patients.

## 7. Pediatric Pain Syndromes: Pathophysiology, Assessment, and Management of Pain in Children from Infancy through Adolescence

### 7.1. Infants and Young Children

Many early studies of SCD in infants and young children concentrated on the initial manifestations of SCD that might be a hint to early diagnosis of this disorder, while subsequent studies after the advent of newborn screening have focused on factors associated with specific clinical events, such as pain, which did not suffer the ascertainment bias that was common in early studies. Bainbridge et al. described the experience from Jamaica of 314 children with SS identified over an 8-year period from 6/73–12/81 [391]. Occurrences of clinical events were recorded retrospectively from information obtained at scheduled clinic visits. No specific sickle cell-related symptoms were recorded prior to 3 months of age, and only 16 (6%) infants had such symptoms prior to 6 months of age. Of these initial symptoms prior to 6 months, 50% were episodes of dactylitis, which was similar in frequency to those over the first 2 years of life. After 2 years of life dactylitis became less common, and more generalized painful episodes predominated. The same patient cohort (233 children from 6/73–8/79) had been used previously to more closely describe the dactylitis episodes [392]. The frequency of episodes was relatively constant between 3 and 15 months and accounted for about half of the dactylitis episodes in the first 5 years of life in SS patients. There was no gender difference in dactylitis frequency. The level of fetal hemoglobin was significantly lower, and the reticulocyte count was significantly higher at 6 months of age in those children with dactylitis, but these differences were no longer significant at 24 months of age. Factors that predict the incidence of dactylitis and painful episodes have also been analyzed using survival curve methods and proportional hazards regression in the Jamaican newborn cohort data [393] concentrating on the age to first occurrence. Patients were grouped into one of three equal groups based on their fetal hemoglobin level at age 5 years, which was similar in rank to fetal hemoglobin levels from 6 months, 2 years, or 10 years of age instead. The trend in occurrence of dactylitis by fetal hemoglobin grouping was highly significant for male, but not female SS children. However, the low fetal hemoglobin group for both males and females had a significantly earlier onset of dactylitis. Similar relationships were seen for painful episodes.

Gill et al. reported the experience of the Cooperative Study of Sickle Cell Disease (CSSCD) which enrolled 694 infants identified with SCD by newborn screening prior to 6 months of age and followed them prospectively up to age 10 years, from 10/78 to 10/88 [394]. Dactylitis resulting in a hospital-based encounter (clinic, ER, or in-patient service) had a peak incidence during the 6–12-month period and was rarely seen after 3 years of age. In contrast, painful episodes not involving the hands and feet were rare in the first year of life and became progressively more common thereafter. Dactylitis was extremely uncommon in SC patients, and painful episodes ranged from about 1/3 to 2/3rd as common as similarly aged SS patients. Overall pain rates were estimated at 0.3 episodes per patient per year [395].

### 7.2. School-Aged and Adolescents

The most comprehensive study of pain frequency in this age group comes from the Cooperative Study of Sickle Cell Disease (CSSCD), which examined a 10-year longitudinal cohort of about 4000 adults and children [395]. Rates of hospital-treated pain averaged in the 5–19 between 0.5 and 1.0 episodes per patient-year. However, there was considerable interpatient variability as 46% of the patients with Sickle Cell Anemia aged 0–9 years, and 32% aged 10–19 years had no episodes in a given year, while 7% of the patients aged 10–19 years had between 3 and 10 hospital visits for pain treatment each year. This latter group of patients accounted for a substantial proportion of all hospital-treated episodes. Higher pain rates were associated with higher hematocrit levels and lower fetal hemoglobin levels on multivariate regression. Similar relationships between pain frequency and hemoglobin levels, fetal hemoglobin levels, and measures of sickle cell adhesion have also been documented in school-aged children and adolescents in the home setting [396]. Recent studies have also suggested a role for the influence of inflammatory mediators in the frequency of vaso-occlusive pain frequency [397, 398].

Health-related quality of life and its relationship to pain have been studied QOL using the PedsQL version 4.0 generic core and fatigue scales in 1772 subjects (53% boys) with a mean age of 9.6 years (SD 4.7) from the Collaborative Data (C-Data) Project of the Comprehensive Sickle Cell Centers (CSCC) Clinical Trial Consortium [399]. Multiple regression models controlling for hemoglobinopathies, gender, and age showed that parent reports of physical functioning and sleep/rest fatigue declined in response to the occurrence of pain or avascular necrosis, while school functioning scales declined in response to pain or asthma. Sickle pain, and to a lesser extent asthma, negatively influenced child reports on almost all functioning and fatigue scales. These and other epidemiologic studies of sickle continue to help us understand the variability of the sickle pain experience. Future studies are necessary to understand the frequent transition from acute recurrent pain in childhood to persistent pain in adults with SCD [400].

### 7.3. Assessment

A substantial literature exists that describes pain characteristics in school-age children (>6 years) and adolescents, and recommendations have been made for appropriate pediatric assessment instruments [401, 402]. Pain intensity assessment in children and adolescents with SCD is similar to other pediatric populations and has largely relied on traditional self-report measures (e.g., visual analogue scales, categorical scales, etc.). Pain assessment in young children becomes increasingly problematic as their cognitive understanding limits their ability to discriminate relative sizes or line lengths or abstract concepts of pain and its causation. Ultimately, self-report fails in children under 3-4 years of age; behavioral observations have been used as alternative measures [403], as has parent-proxy reporting [404].

Initial studies of hospitalized children and adolescents with sickle pain examined 17 adolescent and young adult patients who were hospitalized for treatment of severe pain with parenteral morphine using the Varni-Thompson PPQ [405]. Pain intensity was very high in the first several days of hospitalization (8-9/10) and progressively decreased during the remainder of the hospital stays (*P* < .0001, by ANOVA with hospital day as independent variable and VAS as dependent variable). A similar temporal trend for pain relief was also seen, with generally very poor pain relief early in the painful episode and good pain relief by the time of discharge. Patients reported a mean of  3 ± 2  (mean ± SD) painful sites on day 1 of hospitalization, with spine, sternum, both knees, and both ankles being the four most common sites. Subsequent studies have documented similar findings [406, 407].

A number of studies have described the characteristics of vaso-occlusive pain in the home setting [396, 408]. In a study of 39 children and adolescents followed with daily diaries [396], the number of pain episodes reported in the 6-month study period varied widely between patients (range 0–31 episodes). Patients with at least one episode a month represented 40% of the study participants, while 12% had more than 2 episodes per month. The frequency of painful episodes managed at home was highly variable among patients and did not vary significantly by gender, age, or hemoglobinopathy. Most episodes were relatively brief with 59% of episodes in younger children (5–9 years) and 46% in older children lasting one day or less. On multivariate analysis, older age (10–19 years), female gender, and SCD-SS genotype were associated with significantly longer episode durations. Episodes of one day or less were much more common in individuals with SCD-SC genotype (67%) compared to those with SCD-SS (40%).

Vaso-occlusive pain experienced during these episodes was of mild-to-moderate intensity on the majority of reported days with pain, but severe pain, rated as 7 or higher on a 10-point intensity scale, was reported on 12% of days. Gender or hemoglobinopathy did not significantly influence pain intensity, while younger children on average reported less intense pain than did older children. Pain was localized to one or two distinct sites on 65% days with vaso-occlusive pain. While age and hemoglobinopathy did not influence the number of sites, female gender was associated with a higher number of reported painful sites.

A number of personal factors can also contribute to the sickle pain experience. For example, a study of adolescents with SCD (*n* = 37; aged 13 to 17 years), who completed daily diaries assessing pain, stress, mood, activity, and health-care use for up to 6 months, showed that daily increases in stress and negative mood were associated with increases in same-day pain, health-care use, and reductions in school and social activity using multilevel regression modeling [409]. Increases in positive mood were associated with decreases in pain, less health-care use, and more activity participation. Similar studies suggest that optimism is a significant moderator of the relation between pain and opioid-medication use in adolescents [410]. At medium and high levels of optimism, pain was positively related to opioid use, but at low levels of optimism, the same relation was not present, suggesting that more optimistic adolescents are better able to match their medication use to their pain severity. Self-esteem of adolescents with SCD has also been correlated with less depression and anxiety, while a sense of inadequacy has been associated with poorer functioning [411]. Current and future research is examining how other psychosocial factors might influence pain severity or medication use in adolescents who experience pain. Determining how resilience factors protect adolescents with SCD may aid in the development of future psychosocial interventions and help clinicians understand how to take into account psychosocial factors when working with pain patients.

Another evolving area of pain assessment is the development of assessment tools to characterize the impact of pain on daily activities. The NIH-sponsored Patient Reported Outcomes Measurement Information System (PROMIS) has developed validated pediatric assessment tools for a number of relevant domains including physical functioning and pain interference [412]. Other investigators have developed measures that characterize the ability to perform specific common physical activities that may be impaired by pain [413, 414]. Novel technologies are also being developed to provide objective measures of physical activities [415] that may be useful in characterizing the consequences of pain. These assessments may help guide both research and therapies for acute or persistent pain.

### 7.4. Management

Pain management in children with SCD presents unique challenges and opportunities. A recurrent pain condition with unpredictable occurrence and severity with variable response to pharmacologic therapies represents the fundamental challenge. The opportunity afforded by SCD is to provide anticipatory and concurrent guidance to children and their families while the child is asymptomatic to encourage and support the use of a variety of cognitive-behavioral therapies, positive coping skills, and an acceptance of lifelong pain disorder.

Pharmacologic therapies for acute pain in childhood are similar to that of other acute pain disorders in children. Much of the pain experienced and treated at home is of mild-to-moderate intensity and responses well to acetaminophen or nonsteroidal anti-inflammatory agents (NSAIDs) [416], while more severe acute pain often responds to some degree to a combination of NSAIDs and oral opioids. When adjusted for body weight or size, the pharmacology of commonly used opioids, such as oxycodone, in children older than 6–12 months of age is similar to adolescents or adults [417]. A variety of cognitive-behavioral therapies are likely effective adjunctive therapies for all acute pain [418, 419]. Physical therapies such as heat and massage are also commonly used by children and their families for sickle cell pain [416, 420] and likely effective [421, 422].

Management of severe vaso-occlusive pain in the acute care or hospital setting is somewhat more problematic. Parenteral opioids given on a time-contingent basis, often in combination with parenteral NSAIDs, is the most common pharmacologic therapy in this situation, but few large controlled trials are available to guide dosing. Frequent dose titration to adequate pain relief in specialized day hospital units has been quite effective in children [423], as it has been in adults, but has been difficult to apply to more traditional emergency department settings. Continuation of parental opioids and NSAIDs after admission is common practice, often using patient-controlled analgesia systems in older children and adolescents [406], but large randomized studies are not available to provide an evidence base for practice. Adequate pain management can be compromised by the occurrence of tolerance or hyperalgesia, potentially by excessive dosing of opioids.

Although there are few studies documenting the prevalence of persistent or “chronic” pain in children with SCD, most clinical experience suggests that condition is infrequent in children, but does occur in adolescents. However, there are no controlled trials of pharmacologic therapies for adolescents with SCD and persistent pain. There is experience with cognitive-behavioral therapies for other persistent pain conditions in adolescents [424], and cognitive-behavioral therapy (CBT), relaxation therapy, and biofeedback all produce significant and positive effects on pain reduction, so this experience suggests their likely effectiveness in adolescents with persistent sickle pain. Because traditional behavioral and CBT interventions center on the development of pain coping skills, they may be biased toward a primary focus on pain relief. Acceptance and commitment therapy (ACT), as an extension of traditional CBT, emphasizes the importance of accepting pain symptoms and working toward valued goals, using interventions such as exposure, cognitive defusion, and mindfulness, deemphasizes pain relief and focusing goals toward enhancing daily functioning, and may be more appropriate in the setting of persistent pain [425].

## 8. Adult Pain Syndromes

Pain dominates the clinical picture of SCD in general and sickle cell anemia in particular. Many of the complications of sickle cell disease are associated with either acute or chronic pain. Acute chest syndrome and hepatic cholestasis, for example, are associated with acute pain. Leg ulcers and avascular necrosis, on the other hand, are often associated with chronic pain. Management of these types of pain is associated with the management of the complication in question as will be discussed below under specific organ/tissue damage. This section will address sickle cell pain that is not associated with complications of the disease but itself is a major complication of SCD.

Sickle cell pain is often classified as either acute or chronic. The acute type is the recurrent painful crisis that often requires treatment in the emergency room, day unit, or hospital with parenteral analgesics, mostly opioids. Pain that occurs between the acute episodes is usually milder and treated at home with oral analgesics and is often referred to as chronic pain. Although this classification is somewhat arbitrary, nevertheless management of sickle pain is based on these assumptions as will be discussed below.

### 8.1. Acute Pain

The acute sickle cell painful crisis (also known as acute painful episode or event) is the hallmark of sickle cell disease in both children and adults. It is the number one cause of hospital admissions in adults with 95% of admissions due to acute painful crises [426]. The painful crisis often precedes the onset of other complications of the disease such as acute chest syndrome and acute multiorgan failure. About 50% of cases of acute chest syndrome occur in patients a few days after admission to the hospital with acute painful crises [427]. Moreover sudden death is known to occur during a painful crisis or shortly after discharge from the hospital [428, 429].

The frequency of acute pain crises in patients varies within and between individuals from rare occurrences during a lifetime to many times a month [430]. About 30% of patients have rare or no pain episodes, 50% have occasional episodes, and 20% have weekly or monthly episodes requiring medical attention [431]. The frequency of pain episodes increases late in the second decade of life and decreases in frequency after the fourth decade for reasons that are not understood [430, 432]. Frequency of more than 3 episodes a year is associated with a reduced life expectancy [432]. A small number of patients account for the majority of patients requiring healthcare for acute pain episodes [432].

There are at least three sets of known predisposing events that seem to predict the frequency and severity of the acute sickle cell painful crisis. These are genetic, cellular, and environmental/epigenetic factors.

Genetic factors include Hb F level, the coinheritance of thalassemia (alpha or beta), the coinheritance of other hemoglobin variants (such as Hb C), beta haplotypes, epistatic gene modifiers, and gender. The higher the level of Hb F the milder is the disease and the less frequent are the painful crises [433–435]. The coinheritance of alpha thalassemia, beta^+^ thalassemia, and Hb C decreases the frequency of crises. The Senegalese beta haplotype seems to be associated with less crises than the Benin or the CAR haplotypes [436]. Females are admitted less frequently to the hospital than males but they have longer hospital stay [426, 437].

Cellular factors associated with decreased RBC deformability and increased number of dense cells in the steady state have salutary effect, most likely because these are associated with more severe anemia and, hence, relatively decreased whole blood viscosity [438, 439]. Patients with sickle cell anemia and relatively high hemoglobin levels are more likely to experience more frequent crises than those patients with lower Hb levels [440].

Nocturnal hypoxia, sleep apnea, and nutritional factors such as vitamin A deficiency are environmental factors amenable to preventative therapy [441, 442].

Patients relate onset of pain to emotional stress, changes in weather, exposure to cold, dehydration, infection, fatigue, and overexertion. Major reported factors that seem to precipitate acute painful crises include dehydration, stress of any kind (physical, traumatic, physiological, psychosocial, emotional, etc.), infection, asthma, acidosis, sleep apnea, climate, and pregnancy [443, 444]. Nevertheless, most painful episodes are not preceded by an obvious precipitating factor. Moreover, daily mood and stress predict painful events, utilization of healthcare facilities, and work activity in adults with SCD [445].

Anecdotally, many patients report that sudden changes in temperature seem to precipitate acute painful episodes. Several studies have found an increased incidence of pain episodes during cold and rainy weather [446–448]. Others have disputed this association although these studies were underpowered because of sample size [449–452]. The effect of high wind and low humidity is likely to be related to skin cooling. More recent studies suggest that windy dry weather and increased air pollution may precipitate pain episodes in London [453, 454]. The suggestion by many patients that swimming or exposure to cold water is a precipitant was directly supported in one study [455].

Pain of the acute crisis is often described as throbbing, sharp dull, or stabbing in decreasing order of frequency. Anatomic sites most affected include the back, legs, knees, arms, chest, and abdomen in decreasing order of frequency [456]. The intensity of the pain of the acute crisis is typically >6 on a visual analogue scale, or similar other scales, where 0 denotes absence of pain and 10 indicates the most severe pain [426, 456].

### 8.2. Chronic Pain

Chronic pain is pain that does not go away and lasts for months and even years throughout the lifespan of the patient. It is usually described as deep, nagging, and achy in nature that is there all the time. It could be in the chest, back, abdomen, extremities, neck, or headache. It resides there without invitation and resists any trial for its resolution. It usually follows frequent recurrent and severe acute painful crises. Early and aggressive interventions in treating acute sickle cell pain may be successful in reducing the development of chronic pain. The principles of treating chronic pain are different than those of acute pain. The goal of managing acute pain is to heel the acute injury or precipitating factor(s). The goal of treating chronic pain is to restore function. In a sense chronic sickle cell pain is a spin off the recurrent acute painful episodes. As such it becomes a disease by itself due to neuroplasticity of the central nervous system and central sensitization. To that end, ambient environmental stimuli that are normally innocuous become painful to the sensitized patient with chronic pain.

Once chronic pain sets in it is joined by other maladies that enhance its chronicity. These include depression, anxiety, suffering, despair, insomnia, loneliness, helplessness, and dependence on pain medications. With the onset of chronic pain there seems to be a process of “re-wiring” the brain where the threshold for pain perception is lowered so that ambient environmental stimuli that are normally painless or mildly painful induce the perception of severe pain. Hyperalgesia in this context is the perception of severe pain generated by stimuli that are normally mildly painful, and allodynia is the perception of severe pain by stimuli that are normally painless [443, 457].

**Neuropathic Pain**

It is usually described as numb, tingling, lancinating, shooting, or paroxysmal in nature associated with a sensation of pins and needles. Its severity is also enhanced by exposure to either cold or heat. This pain was believed to be secondary to nerve injury whether peripherally or centrally. This definition has recently been modified by the International Association for the Study of Pain (IASP) to include injury and dysfunction of nerves as causes. Persistent chronic pain and/or its management seem to cause the perception of neuropathic pain. The pathophysiologic events leading to this transformation are not well understood also. Activation of the glia seems to be a possible pathophysiologic mechanism leading to neuropathic pain [458].

**“Breakthrough” Pain**

It is another kind of pain often referred to by care providers following patients with SCD. Literally this term means the act of breaking through pain relief. Originally this term was introduced for patients with cancer pain [459] and was defined as a flair of sudden [457] pain in patients with cancer pain that were maintained on a stable dose of oral analgesics to achieve adequate relief. Such flair is usually sudden and incidental, precipitated by movement, and may last from a few seconds to a few hours. Recommended management includes the administration of short-acting analgesics via a route that achieves immediate relief such as parenteral or transmucosal routes. Details of the definition and management of breakthrough pain are controversial to date, and its application to other types of pain seems arbitrary. Health care providers treating patients with sickle cell pain use the term “breakthrough” pain loosely for any pain that occurs in patients taking analgesics daily for chronic pain and treat it with short-acting oral analgesics that are the same or different than the long-acting analgesics the patients are already taking. Moreover the short-acting analgesic is often given several times a day in cumulative excessive doses that are as high as or even higher than the daily dose of the long-acting opioids the patients are already taking. Thus this kind of practice is not for breakthrough pain but rather an administration of extra or rescue doses of analgesics for pain that is inadequately treated in the first place and implies that the dose of the long-acting analgesic used should be increased to achieve better pain relief.

**Other Types of Pain**

They include postoperative pain and iatrogenic pain due to therapeutic interventions. These are not unique to SCD and, hence, will not be discussed in this paper.

### 8.3. Management

Effective management of acute pain in the emergency room, day unit, or in the hospital depends on systematically following certain sequential steps (Table 3) that start with thorough assessment coupled with the utilization of nonpharmacologic and pharmacologic modalities of therapy culminating in a plan for discharge and followup.

### 8.4. Assessment

Assessment is the basis of effective pain management. It should be done before and periodically after the initiation of analgesic therapy [443, 460, 461]. The patient's self-report is the most important factor in the hierarchy of pain management. Other factors in the process of assessment should include the presence or absence of other complications of the disease, such as infection, family members report, and vital signs, including temperature, blood pressure, pulse, respiratory rate, and pulse oximetry. The patient's self-report should include multidimensional scales describing intensity, quality, location, distribution, onset, duration, mood, sedation, pain relief, and factors that aggravate or relieve pain [460, 462].

The intensity of pain can be assessed by any of several available scales, such as the visual analogue scale, verbal scale, numerical scale, or Wong-Baker faces scale for children. It is important, however, to stick to one scale and use it routinely, so that both the patient and provider become familiar with it and with its significance to a particular patient. Nociceptive sickle cell pain typically is sharp or throbbing in nature. Pain that is burning, shooting, lancinating, or tingling suggests the presence of a neuropathic component that entails the use of certain adjuvants mentioned below [443, 460].

#### 8.4.1. Nonpharmacologic Management of Pain

Table 4 lists the various approaches to nonpharmacologic therapy. It is important to note that these approaches do not apply to all patients all the time. Certain methods may appeal to some but not all patients. Counseling the patient in order to determine which nonpharmacologic method the patient chooses would be associated with the most desirable outcome. Although there are no well-controlled clinical trials of the efficacy of these methods in the management of sickle cell pain, there are many anecdotal reports of their efficacy in pain management.

#### 8.4.2. Pharmacologic Management of Pain

Pharmacologic management of pain includes three major classes of compounds: nonopioids, opioids, and adjuvants [443, 460, 461]. A major difference between nonopioids and opioids is that the former has a “ceiling effect,” a term that refers to a dose above which there is no additive analgesic effect [457, 458, 463]. Nonopioids include acetaminophen, nonsteroidal anti-inflammatories (NSAIDs), topical agents, tramadol, and corticosteroids.

#### 8.4.3. Opioid Analgesics

These compounds have fewer systemic adverse effects than NSAIDs [464], but their use in SCD is associated with many myths about drug-seeking behavior and addiction. Four major classes of opioids there exist agonists, partial agonists, mixed agonists-antagonists, and antagonists (Table 5).

Opioid agonists are most often used in the management of sickle cell pain. They decrease or modify the perception of pain at the level of the central nervous system. They exert their effect by binding to *μ*-, *κ*-, and, to a lesser extent, *δ*-receptors [457, 458, 464]. Opioid agonists can be administered by several routes (e.g., orally, subcutaneously, intramuscularly, intravenously, transdermally) and methods, including continuous intravenous drip, patient-controlled analgesia (PCA) pump, and intermittent injection. Morphine and hydromorphone are the major opioid analgesics used in the treatment of severe acute pain in the emergency department, day unit, and hospital. Controlled released opioids, such as controlled-release (CR) oxycodone and morphine CR, are useful in the management of chronic pain and in combination with short-acting opioids for breakthrough pain. Fentanyl is available in parenteral, transdermal, and transmucosal formulations. Methadone is a true long-acting opioid that can be used in combination with short-acting opioids in selected patients.

Adverse effects of opioid analgesics include itching, nausea, vomiting, sedation, and respiratory depression. Seizures may be associated with opioids, especially with the prolonged use of meperidine (pethidine) and the consequent accumulation of its major metabolite, normeperidine, in some patients. The effects of meperidine and normeperidine on seizure induction are more pronounced in the presence of renal disease. Tolerance and physical dependence occur in some patients, but addiction is rare [430]. Methadone may be associated with prolongation of the QTc interval [465].

Opioid analgesics have no ceiling effect (with the possible exception of codeine); hence the only limiting factor on their dose is adverse effects. Severe sedation and respiratory depression are the most important adverse effects. Hospitalized patients receiving opioid analgesics on a regular basis should be monitored for their respiratory rate and sedation level. A respiratory rate of less than ten per minute or severe sedation justifies skipping, decreasing, or delaying the dose or discontinuing the opioid in question until the depressive effects disappear. Opioid analgesics should be used carefully in patients with impaired ventilation, asthma, increased intracranial pressure, and liver failure. The presence of acetaminophen in combination with codeine or oxycodone limits the daily dose that may be safely used so that the maximum allowable dose of acetaminophen is not exceeded. The FDA has recently decreased the maximum allowable daily dose of acetaminophen to 3000 mg/day instead of the previous 4000 mg/day [466].

Providers who prescribe opioids for patients with sickle cell pain must be cognizant of the current legal requirements. Specifically and besides prescribing enough opioids to optimize pain relief, providers must institute measures to minimize the risk of misuse, abuse, or diversion of the prescribed opioids. Specific approaches to these recommendations have been published by the American Pain Society in collaboration with the American Academy of Pain Medicine [467, 468]. Most important among these include the establishment of a consent form, treatment plan, random urine drug testing and thorough documentation of the plan of management, and followup of each patient who receives opioid prescriptions on a regular basis.

**Adjuvants**

They include antihistamines, antidepressants, benzodiazepines, and anticonvulsants. These are heterogeneous compounds that potentiate the analgesic effect of opioids, ameliorate their side effects, and have their own mild analgesic effect. The role of selective serotonin reuptake inhibitors in sickle cell anemia is not clear at present. Adjuvants must be used with care, and patients should be monitored carefully when receiving them. Adjuvants also have adverse effects, some of which precipitate or worsen manifestations of sickle cell anemia [430].

Acute painful episodes of mild or moderate severity are usually treated at home using a combination of nonpharmacologic and pharmacologic modalities. Home treatment of pain usually follows the three-step analgesic ladder proposed by the World Health Organization [469]. Mild pain is treated with nonpharmacologic agents alone or in combination with a nonopioid. More severe pain entails the addition of an opioid with or without an adjuvant.

Patients with chronic sickle cell pain are best managed with a combination of long-acting opioids and a short-acting opioid for breakthrough pain. Again, anecdotal reports suggest that this approach decreases the frequency of admissions to the emergency department or hospital, but data to confirm this finding are not available to date. Oxycodone CR appears to be unique in that it has both an immediate analgesic effect and a delayed long-acting one. These properties have made oxycodone CR popular among drug abusers who have learned to remove the mesh and release a high dose of pure oxycodone that has an immediate “euphoric” effect [470]. Care providers should exert caution in prescribing oxycodone CR and other opioids and should keep detailed records of assessment and plans of management of their patients.

#### 8.4.4. Preventative Therapies

Measures to reduce the morbidity and mortality of sickle cell anemia include prophylactic penicillin therapy in infants and children [471] and hydroxyurea [472–474]. Although hydroxyurea was first approved for adults with sickle cell anemia and sickle-*β*0-thalassemia, results of the recently described Baby HUG trial justify its use in children as well [475]. Patients who responded to hydroxyurea experienced significant reduction in the incidence of acute painful episodes, acute chest syndrome, transfusion requirement, and mortality [473, 474, 476]. The beneficial effects of hydroxyurea are thought to be due to its induction of Hb F production. Any increase in Hb F level appears to have a salutary effect on the clinical picture of sickle cell anemia.

## 9. Pulmonary Complications

### 9.1. Acute Chest Syndrome

The clinical manifestations of acute chest syndrome (ACS) complicating SCD include chest pain, tachypnea, fever, hypoxia, dyspnea, cough, leukocytosis, decreasing Hb level, and new infiltrates on chest X-ray [477–479]. Not all these signs and symptoms occur in all cases of ACS with the exception of the new pulmonary infiltrates which are considered the *sine qua non* for the diagnosis. The presence of new infiltrates with some of the other signs and symptoms is usually enough to make the diagnosis. An infiltrate is new when compared to a previous radiograph with no infiltrate. If a previous radiograph is not available, the infiltrate in question is considered as new. It is obvious from this description that there are gaps in making an accurate diagnosis. For example, there is no agreement on the number and nature of the accompanying signs and symptoms to make the diagnosis. Moreover, an occasional patient may have all the signs and symptoms mentioned above with no new infiltrate on chest radiograph thus generating a dilemma for the provider. Suffice to say is that the ACS, like other syndromes, is a spectrum of clinical manifestations that vary from the mild to the very severe. Observation and careful monitoring on a daily basis or more often if needed are most important in ruling the diagnosis in or out.

The incidence of ACS is age and genotype dependent with no difference between the sexes. It is about three times more common in young children than in adults but more severe in adults [478, 479]. ACS is most common in SS, S-*β* ^0^-thalassemia, Hb SC disease and S-*β* ^+^-thalassemia in decreasing order of frequency. Coexistent *α* gene deletion, the platelet count, and the mean corpuscular volume (MCV) of RBC do not seem to affect the incidence of ACS [478]. The incidence of ACS decreases in the presence of high Hb F level and severe anemia but is directly proportional to the steady-state WBC count [478]. ACS is closely associated with acute painful crises especially in adults [480, 481]. About 50% of ACS episodes occur after hospital admission for acute painful crises [480]. Moreover, ACS seems to be the most common cause of death among patients and the second most common cause of hospitalization of patients with SCD [482–485].

Causes of ACS include pneumonia, bone marrow fat embolism, pulmonary infarct due to *in situ* sickling, rib/sternal infarction, infection, and pulmonary embolism [480, 486–488]. About 50% of patients with ACS have no identifiable etiology [480]. Rib or sternal infarction leads to hypoventilation due to splinting with consequent atelectasis and retention of secretions that may initiate an infectious process [486, 487]. Infectious etiologies of ACS include typical and atypical bacteria, typical and atypical viruses, or other organisms. Specific infectious etiologies include chlamydia, mycoplasma, respiratory syncytial virus, coagulase-positive *S. aureus*, *S. pneumonia*, *Mycoplasma hominis*, parvovirus and rhinovirus in decreasing order of frequency [480]. *In situ* sickling leading to *in situ* thrombosis/pulmonary infarct is different from the usually known PE due to emboli from the lower extremities or pelvis. The prevalence of apparent PE in patients with SCD is higher than in non-SCD African-American patients with the same age despite the fact that the prevalence of DVT was comparable in both groups [489]. This discrepancy suggests that the apparently surplus PE in patients with SCD is most likely due to *in situ* thrombosis. This differentiation is important in deciding whether long-term anticoagulation is indicated or not.

Prospective multicenter study of ACS in SCD showed that bone marrow/fat embolism is more common than previously thought [480]. Severe sickle cell pain in long bones followed by dyspnea, hypoxia, and fever is the clinical picture suggestive of fat embolism. Tissue infarction of the bone marrow within long bones seems to be the source of fat and necrotic tissue that finds its way to the blood stream and from there to the lungs, brain, or other organs. The diagnosis of fat embolism entails the identification of fat-laden macrophages (fat bodies) in blood, urine, induced deep sputum, or, better, in bronchoalveolar lavage fluid obtained by bronchoscopy [490].

Management of ACS utilizes multiple modalities in order to prevent possible catastrophic outcomes. The most important aspect of management is to maintain adequate ventilation. In mild cases incentive spirometry may be sufficient to achieve this. In severe cases, however, mechanical ventilation in the intensive care unit is essential. Once adequate ventilation is maintained, specific treatment includes oxygen, antibiotics, simple blood transfusion or exchange transfusion, judicious use of analgesics, bronchodilators, careful hydration, and possible vasodilators. Incentive spirometry prevents splinting and atelectasis and may actually prevent ACS in patients who have rib infarction [487]. Intravenous antibiotics are indicated since it is difficult to rule out pneumonia or infected lung infarcts. A combination of a third generation cephalosporin and a macrolide or a quinolone antibiotic should be used to cover typical and atypical pathogens. Simple transfusion or exchange transfusion is indicated in patients with worsening respiratory function [491]. The beneficial effects of blood transfusion may not be due simply to decreasing the proportion of sickled RBCs, and other mechanisms may be involved. These include (1) an immunomodulatory mechanism by which inflammatory cytokines (IL-8 in particular) bind to the Duffy antigen present on transfused RBCs, but often absent on RBCs of African-Americans [490]; (2) the albumin that is present in transfused units or used in blood exchange may bind free fatty acids, thus neutralizing their damaging effect on the pulmonary endothelium.

Although intravenous steroids in children with ACS may be beneficial [492], their use in adults with ACS is controversial. Huang et al. [493] reported two adult patients with sickle cell disease whose clinical picture deteriorated and was complicated by worsening pain, fat embolism, and coma after steroid therapy. Other investigators had similar experience with steroids. [494, 495] Adults, unlike children, have more adipose tissue that may hypertrophy with steroids, increasing the chances of fat embolization. Moreover, steroids may induce or worsen avascular necrosis which is more common in adults than in children. Management of ACS due to proven PE includes anticoagulation as used in PE in general. The role of anticoagulation in patients with ACS due to *in situ* thrombosis is unknown.

Excessive opioid analgesics may precipitate acute chest syndrome due to their depressive effect on respiration. This recommendation should be considered carefully. Opioids have a few systemic side effects, and careful monitoring of their use ensures their safety. They should be discontinued if the respiratory rate is ≤10/minute and their adverse effects can be quickly reversed with opioid antagonists. NSAIDSs, on the other hand, have considerable systemic side effects that may not be readily obvious. NSAIDSs decrease the levels of prostaglandins, prostacyclin, and prostanoids that are essential in modulating the vascular tone of smooth muscle and renal blood flow. Thus, NSAIDSs may worsen the clinical picture of ACS due to their vasoconstrictive effects and bronchospasm. NSAIDSs are contraindicated in asthma for the same reasons.

The use of nitric oxide, a vasodilator, in patients with SCD supports a possible role of this agent in the management of ACS [496]. Other vasodilators such as prostacyclin and calcium channel blockers have not been reported in the management of ACS. Another recent investigational approach to treat ACS includes the use of purified poloxamer 188 (Flocor), which is a nonionic surfactant [497, 498]. It is hypothesized that this agent reduces blood viscosity, prevents adhesion of RBCs to vascular endothelium, and improves microvascular blood flow.

The role of vasodilators in the management of SCD in general and ACS in particular is not finalized. Nitric oxide (NO) had a beneficial effect on painful crises in children [499] and in adults in the emergency room setup [500, 501]. It use on hospitalized patients with painful crises had no beneficial effect [502]. An open trial using purified poloxamer 188 (Flocor), a nonionic surfactant, in patients with ACS showed no benefit [497]. Other vasodilators such as prostacyclin and calcium channel blockers have not been reported in the management of ACS.

## 10. Pulmonary Hypertension

### 10.1. Prognosis and Prevalence of PHT in SCD

Pulmonary hypertension (PHT), which is defined as *mean* pulmonary artery pressure (PAP) of 25 mm Hg, determined by right heart catheterization (RHC), is one of the leading causes of mortality and morbidity in adults with sickle cell disease (SCD). Interestingly, elevated *systolic *PAP, derived from echocardiography-based tricuspid jet velocity (TRJV), if present in adult patients with SCD, also portends a poor prognosis.

Castro and colleagues were amongst the first to study sickle PHT and determine the relationship of PHT (determined by RHC) to patient survival. They studied 34 adult patients with SCD and showed that mean PAP had a significant inverse relationship with survival, with each increase of 10 mm Hg in mean PAP associated with a 1.7-fold increase in the rate of death; the median survival for patients with PHT was 25.6 months, whereas for patients without PHT, the survival was >70% at the end of the 119-month observation period, showing that onset of PHT in patients with SCD significantly shortens their survival [503]. Gladwin et al. subsequently reported that elevated systolic PAP (derived from echocardiography based TRJV ≥2.5 m/s) strongly correlated with an increased risk of death (odds ratio, 10.1); the risk of death increased significantly with TRJV >3 m/s [504]. Recently, two large studies in patients with SCD have determined that PHT (determined by using RHC, the gold standard for diagnosis of PHT) is associated with poor survival of adult patients with SCD [505, 506].

Following the report by Gladwin et al. [504], TRJV >2.5 m/s (or increased peak/systolic PAP) became erroneously synonymous with sickle PHT for the next 6 years, until some recent critical reviews of the literature [507, 508] and two recent reports on RHC on patients with TRJV >2.5 m/sec [505, 506] which highlighted the fact that while TRJV ≥2.5 m/s may be uniformly associated with poor outcome in adults with SCD, it is not equivalent to PHT in the majority of patients that have a high TRJV (>2.5 m/s); the predictive value of TRJV is about 25–33% [505, 506].

PHT has been reported to be present in 6–33% of adult patients with SCD in different studies, with most studies using echocardiography-based TRJV to predict PHT. It is becoming increasingly clear that echocardiography-based studies (tricuspid regurgitation jet velocity; TRJV) overestimate the prevalence of PHT in nearly two-thirds of adults with SCD [504], while RHC data suggest a lower prevalence in 6–10% of patients [505, 506]. *That said, elevated TRJV in adults with SCD, regardless of whether they have RHC-proven PHT or not, predicts a poor survival.* Hence high TRJV is likely indicative of other underlying cardiac pathologies, such as ventricular diastolic dysfunction, vasculopathy/endothelial dysfunction, or perhaps a sickle cardiomyopathy resulting from the chronic hypoxic stress on the myocardium. Recently, Knight-Perry et al. found cardiac structural and functional abnormalities in patients with high peak systolic PAP (TRJV >2.5 m/s). They assessed RV and LV structure and function via echocardiography in adults with SCD. Patients who had high TRJV (>2.5 m/s) had higher left atrial volumes, LV and RV filling pressures, and reduced LV and RV compliance [509]. More recently, DeBaun and colleagues have reported diastolic dysfunction in patients with SCD [510]. Therefore, other parameters of echocardiography besides TRJV, and a second imaging modality, such as cardiac MRI should be assessed in addition to TRJV.

In children, TRJV defined of systolic PHT is more controversial, where high TRJV ≥2.5 m/s is not reproducible [511]. Pashankar and colleagues conducted a longitudinal followup of 54 children. Only half the children with elevated TRJV showed persistently higher TRJV at followup, and most of those with persistently elevated TRJV were those with TRJV >2.7 m/s [511, 512]. Furthermore, elevated TRJV (TRJV >2.5 m/s) has not been shown to carry the dismal prognosis that is seen in SCD adults with high TRJV. A large prospective study in over 400 children with SCD and age-matched controls found that TRJV up to 2.6 m/s was found in normal children, and TRJV >2.6 m/s in children with SCD was associated with an increased hemolytic index [513]; a followup of 160 of adults and adolescents that had high TRJV or diastolic dysfunction from this cohort showed a higher risk of developing exercise intolerance, as measured by a 6-minute walk distance [514].

### 10.2. Pathophysiology of PHT in SCD

The last six years have seen an upsurge of studies on the pathophysiology of PHT in SCD. In clinical studies, high TRJV is associated with a higher rate of hemolysis, renal insufficiency, leg ulcers, increasing age, low exercise capacity and high levels of N-terminal probrain natriuretic peptide or vascular cell adhesion molecule-1, and a hypercoagulable state [513, 515–522]. Factors that have been implicated in PHT in SCD include endothelial dysfunction, pulmonary vasoconstriction, and vascular remodeling. All of these factors are associated with chronic hemolysis, hypoxia, hemostatic activation, and inflammation [518–520].

The hemodynamic etiology of PHT is multifactorial; studies show that elevated systolic PAP is associated with either pulmonary arterial hypertension, pulmonary venous hypertension, and PHT secondary to a hyperdynamic state associated with right and/or left ventricular hypertrophy and diastolic dysfunction [510, 523–525]. In a recent hemodynamic assessment, patients with SCD-associated PHT (diagnosed by RHC) were found to either have pulmonary arterial hypertension or venous PHT, both associated with severe limitations in exercise capacity in the 6-minute walk test [525]. Myocardial dysfunction is also being increasingly reported in patients with SCA, suggestive of a “sickle cardiomyopathy”, ventricular remodeling and even ischemia [510, 526–530].

Reduced nitric oxide bioavailability has been implicated in the pathophysiology of PHT in SCD and other hemolytic anemias [513, 517, 518, 531]. This has been attributed to reduced argninine levels, from the increased RBC-derived-arginase in SCD [518, 531–533]. ET-1 and nitric oxide (NO) are opposing pulmonary vasoactive factors that regulate pulmonary vascular tone [521, 522]. Studies have shown that hemolysis is associated with PHT in SCA [522]. Hemolysis results in quenching of NO by extracellular hemoglobin thereby reducing the bioavailability of NO [522, 523].

ET-1, a potent pulmonary vasoconstrictor, is elevated in SCA patients with PHT [505, 521, 524]. ET-1 is normally induced in endothelial cells in response to hypoxia as a result of activation of HIF-1*α* [524–526]. ET-1 receptor antagonists, used for the treatment of primary PHT [527], have been found to be beneficial in sickle-Antilles-hemoglobin D mice [528], indicating the important role of ET-1 in PHT in SCA. Malik and Kalra have shown that placenta growth factor, an angiogenic growth factor produced in high amounts by sickle erythroid cells, induces hypoxia-independent expression of hypoxia inducible factor-1 (HIF-1*α*). HIF-1*α* then induces expression of the potent pulmonary vasoconstrictor endothelin-1 (ET-1) [534], and a profibrotic and procoagulant factor, plasminogen activator inhibitor-1 (PAI-1) from human pulmonary microvascular endothelial cells (HPMVECs) [535]. Indeed, ectopic expression of placenta growth factor in normal mice, using a lentiviral vector or an adenoviral vector to generate similar placenta growth factor levels seen in sickle mice, resulted in increased ET-1 and plasminogen activator inhibitor-1 (PAI-1) production and development of PHT, similar to PHT seen in sickle mice [535, 536]. These findings were corroborated by Kato and colleagues in patients with SCD; in these patients, increased plasma placenta growth factor levels were significantly associated with increased plasma ET-1 levels and increased TRJV derived systolic PAP [536]. The association of placenta growth factor and high TRJV was confirmed independently by Ataga and colleagues [537]. Hence, it appears that while there is reduced bioavailability of NO, a potent pulmonary vasodilator, ET-1, a potent pulmonary vasoconstrictor, is increased, resulting in PHT.

A hypercoagulable state and microthrombi have been shown to contribute to PHT in other diseases. SCD is a hypercoagulable state. Studies show that SCD patients have elevated steady-state plasma levels of circulating tissue factor [529] and PAI-1, both of which further increase during sickle vaso-occlusive crises [530, 531]. Elevated PAI-1 has been implicated in primary PHT [509, 510]. The prothrombotic state may predispose patients to PHT and stroke [518, 519, 532]. In addition to the role of PAI-1 in coagulation, PAI-1 also has been shown to be responsible for development of pulmonary fibrosis [533], a feature of PHT. The expression of PAI-1 is also modulated by other stimuli such as hypoxia, TGF-*β*, insulin, and lipopolysaccharide [534–537]. Our group has shown that PAI-1 is directly induced by the erythroid cell-derived placenta growth factor [535].

Recently, albuminuria in SCA has been associated with high TRJV [538], an association which may just be coincidental, since both PH and SN develop with cumulative chronic organ damage with increasing age. The association of albuminuria has also been reported in a longitudinal followup study of children where a higher percentage of albuminuria was observed in the subset of children with high TRJV [512].

### 10.3. Therapeutic Modalities for Sickle PHT

Currently, there are limited data on the effects of any specific treatment modality for PH in patients with SCA. Endothelin-1 receptor antagonists have been tried, are safely tolerated, and show some hemodynamic and functional improvement [526, 539]. Indeed, bosentan was able to reverse PHT in a mouse model of sickle cell disease [540]. However, the clinical trial of bosentan was closed due to inadequate accrual.

A multicenter trial of sildenafil was initiated to improve NO bioavailability (Walk-PHASST) [514, 541]. Here patients with high TRJV were given sildenafil and a detailed assessment of RV function, and exercise tolerance was assessed. Unfortunately, this trial had to be prematurely terminated due to increased painful vaso-occlusive events reported with sildenafil.

Oral arginine supplementation was attempted in a multicenter trial to improve NO bioavailability, but the results of this trial were inconclusive (unpublished data). Chronic transfusions and hydroxyurea are currently being tried for patients with high TRJV/PHT, and the results of these trials are eagerly awaited. Currently, there is no targeted therapy available for patients with SCD that develop PHT. Therefore, newer therapies for sickle PHT are desperately needed.

### 10.4. Summary

PHT is one of the leading causes of morbidity and mortality in adult patients with SCD. However, there are significant gaps in knowledge in the predictors, accurate screening modalities, pathophysiology, and treatment of sickle PHT. PHT is defined as a mean pulmonary arterial pressure (PAP) of ≥25 mm Hg by RHC, but the invasive nature of this modality is prohibitive to using RHC as a screening tool. There is lack of an accurate noninvasive screening test for PHT in SCD. In children, where RHC is rarely performed, data on PHT is much more limited and restricted to echocardiography-based projections, which are less predictive of the prognosis than similar estimations in adults. Echocardiography-obtained tricuspid regurgitation jet velocity (TRJV) is used to estimate peak/systolic pulmonary artery pressure. Recent studies show that high TRJV/Peak PAP only predicts PHT in 25–33% of adult patients with SCD. Nevertheless, high TRJV is associated with poor prognosis in multiple studies, suggesting that additional cardiac/endothelial pathology leads to increased systolic PAP in SCD and portends a poor prognosis. The prognosis of PHT in SCD is poor, with the development of PHT associated with a 40–50% risk of death within 2-3 years (odds ratio of 8–15) [504, 542, 543]. Second, not all patients with SCD develop PHT, and biomarkers that define the subgroup of patients destined to have PHT are not well defined. Hence, novel/refined noninvasive diagnostic modalities and biomarkers are much needed to detect the early onset of PHT in SCD in susceptible patients and timely therapeutic intervention. The pathogenesis of PHT in SCD is likely multifactorial, including hemolysis, impaired nitric oxide bioavailability, chronic hypoxemia, and increased endothelin-1, a potent pulmonary vasoconstrictor, mediated via hypoxia and via the erythropoietic cell-derived placenta growth factor, chronic thromboembolic disease, and asplenia [516]. The hemodynamic etiology of PHT is multifactorial and includes pulmonary arterial hypertension, pulmonary venous hypertension, and PH secondary to a hyperdynamic state associated with right and/or left ventricular hypertrophy and diastolic dysfunction [510, 523–525]. Finally, therapeutic options of PHT in patients with SCD are extremely limited. Clinical trials using bosentan (an endothelin-1 receptor blocker) [539] and sildenafil (Walk-PHASST) [541] had been initiated in patients SCD with PHT, but could not be completed due to lack of accrual in the former trial, and increased painful events secondary to sildenafil, in the latter. Therefore, currently, there is no proven beneficial treatment for SCD patients that develop PHT.

## 11. Renal/Genitourinary Complications

### 11.1. Sickle Nephropathy

#### 11.1.1. Introduction

The kidney appears to get affected in several different ways in sickle cell disease (SCD). Children, and even infants with SCD have hyposthenuria/urine concentrating defect (UCD), supranormal glomerular filtration rate (GFR) and proximal tubular function, and an impaired ability to acidify urine or excrete potassium (reviewed in references [544–547]). A majority of patients with SCD have evidence of microscopic hematuria and may even develop gross hematuria from renal papillary necrosis. Older individuals have been found to have glomerulopathy, that manifests as microalbuminuria (MiA, defined as urine albumin of 30–300 mg/g urine creatinine), macroalbuminuria (MaA, defined as urine albumin >300 mg/g urine creatinine), or end-stage renal disease (ESRD) [544, 545, 548, 549]. Studies show that gross proteinuria and ESRD are observed in 15 to 30 percent of patients with SCD [550, 551]. Kidney biopsies reveal enlarged glomeruli, and the most common glomerular lesion in sickle nephropathy (SN) is focal and segmental glomerulosclerosis (FSGS), while membranous glomerulopathy has also been observed in some cases. We will review the literature on SN with respect to (1) abnormal hemodynamics and hematuria, (2) tubular defects, and (3) glomerulopathy.

#### 11.1.2. Abnormal Hemodynamics

The renal plasma flow and increased GFR are increased in SCA, possibly from the compensatory hypersecretion of vasodilator prostaglandins in response to hypoxia-induced sickling [552]. GFR decreases during adolescence to normal levels, and in older adults, it is subnormal. Inducible nitric oxide synthase (iNOS) is increased in the glomeruli and distal nephrons in sickle mice secondary to hypoxia and is associated with increased urinary excretion of NO products, suggesting that the increased NO-induced vasodilatation also contributes to the increased GFR [553]. There is controversy on the glomerular ultrafiltration coefficient with some studies showing increased glomerular permeability, while others showing reduced ultrafiltration; differences in results in these studies probably reflect sampling of patients at different stages of SN. Chronically increased glomerular hyperfiltration and iNOS-induced peroxynitrite free radical-mediated nitrosylation of renal proteins promote cellular apoptosis, resulting in FSGS [553]. Episodic hypoxia-reperfusion injury superimposed on these renal hemodynamics results in areas of nephron loss and scarring.

**Hematuria**

Vascular obstruction from sickled RBCs leads to microscopic-to-gross painless hematuria, occurring from medullary congestion and renal papillary necrosis. The hypoxic (pO_2_ 35–40 mm Hg), hyperosmolar medullary environment promotes sickling. The vasa recta become congested, tortuous, occluded, and hemorrhage, resulting in painless hematuria. They eventually undergo fibrosis, resulting in infarctive papillary damage and necrosis. Hematuria is often from the left kidney, occurs at any age, and is even seen in persons with sickle trait. Renal papillary necrosis is observed in as many as 40–50% of patients with SCA. Painful hematuria in patients with sickle trait has been associated with renal medullary carcinoma, a very aggressive malignancy characteristically seen in this group of young adults. Damage to the juxtamedullary nephrons and collecting ducts also occurs and contributes to the UCD and to tubular defects.

#### 11.1.3. Tubular Defects

**Urine-Concentrating Defect**

The first manifestation of distal tubular defect is impaired urine concentrating ability, which is almost universal in patients with SCD and occurs in children, even infants. UCD is transiently reversible before 10–15 years of age with RBC transfusions but becomes irreversible thereafter [554, 555]. UCD in SCD has been attributed to polymerization of Hb S in the hyperosmolar, acidic and relatively hypoxic renal medulla, resulting in sludging of blood flow in the vasa recta, loss of medullary osmolar gradient, and eventual destruction of the vasa recta from vaso-occlusions/thrombosis [556]. UCD is associated with increased tendency to dehydration and sickling, enuresis and nocturia [556].

Studies examining UCD, done 5-6 decades back, show inability to concentrate urine either after water deprivation or injection of pitressin in patients 1–30 years of age [554, 555, 557–559]. Interestingly, patients with other sickle variants, hemoglobin SC or S-thalassemia and sickle trait also develop UCD, often with a much later onset than those with homozygous SCD (SS) [554, 558]. Sickle trait with concurrent *α*-thalassemia attenuates the defect, proportional to the reduction in the percentage of hemoglobin S in the cell, with lower Hb S in the RBC being associated with a later onset of UCD [560]. Notably, in children, RBC transfusions can reverse the UCD, but the defect is irreversible after the age of 10–15 yrs [554, 555]. Hydroxyurea therapy results in an increase in fetal hemoglobin production and dramatically reduces the frequency of pain and acute chest episodes in individuals with SCD [561]. However, hydroxyurea showed no improvement in UCD in children with hemoglobin SC disease [561]. A recently completed multicenter BABY-HUG study [562] should provide useful prospective data on the usefulness of hydroxyurea in alleviating the UCD in early SN.

**Potassium and Acid Secretion**

It occurs primarily in the distal tubules and collecting ducts that are present in the renal papillae. Therefore, patients with SCD have impaired ability to excrete acid and potassium [563–565]. Although hyperkalemia does not occur under normal circumstances, patients with SCD are unable to excrete a high potassium load as compared to normal controls [563], and some patients become hyperkalemic when placed on angiotensin-converting enzyme inhibitors. Similarly, hyperchloremic acidosis has been described with mild renal insufficiency [566–568].

**Supranormal Proximal Tubular Function**

In contrast to distal tubular defects (which impair urine concentration and acid secretion), supranormal proximal tubular function is present in SCD, as evident by increased reabsorption of sodium, phosphorus, and increased excretion of creatinine and uric acid in the urine (reviewed in [544, 545]). Hence, plasma creatinine levels are low in patients with SCD and not a good biomarker or indicator of renal function, until late stages of SN, especially since high GFR; creatinine clearance is also present and hypertension occurs late. Since classic renal biomarkers are not informative, novel biomarkers are necessary to monitor for development of SN early in its course, before the damage becomes largely irreversible.

#### 11.1.4. Glomerulopathy

Glomeruli, especially the juxtamedullary glomeruli in young SCA patients, are enlarged and congested, reaching a size that is 60–80% larger than normal glomeruli [546]. Glomerulopathy is associated with albuminuria, with MiA present in 25% of children <10 years of age, and 45% of adults. MaA develops later with progression to FSGS. Glomerular lesions are typically FSGS, mesangial proliferation, endothelial damage and sclerosis from hyperfiltration, immune-complex nephritis from autoantigens released from damaged tubules, and deposition of iron protein complexes in the kidney. A small proportion of patients have membranoproliferative glomerulonephritis with or without immune deposits. The pathogenesis of glomerulopathy is unknown but presumed to occur from mesangial phagocytosis of erythrocytes and apoptotic cells. Macroalbuminuria, especially over 1.5 gm per day, strongly correlates with progression to renal failure, ESRD, and with acute chest syndrome [546, 547, 569, 570].

#### 11.1.5. Biomarkers of SN

Standard renal function tests like serum creatinine and GFR become subnormal in this disease only when renal damage has become extensive and largely irreversible. Therefore, good biomarkers that predict early SN need to be identified. Microalbuminuria is the most studied biomarker of SN in cross-sectional studies. We and others have shown that prevalence of albuminuria in SCD increases with age [570–575]; albuminuria is more commonly seen in patients with Hb SS disease as compared to those with Hb SC or Hb S-*β*+ thalassemia [572]. Guasch et al. have reported that 70% of adults with SCD have albuminuria, while only 40% of adults with other types of sickle cell disease have albuminuria [572]; microalbuminuria data shows the same trend in children, although a lower percentage of homozygous SCD patients have albuminuria [575–578]. Albuminuria could be a useful, albeit delayed biomarker of SN. Studies in diabetic nephropathy suggest that glomerular damage has already commenced when microalbuminuria is detected [579], and not all diabetic patients who have microalbuminuria go on to develop gross albuminuria [580]. Whether microalbuminuria is a predictable biomarker of progressive SN can only be determined by a longitudinal analysis. Notably, albuminuria in SCD has also been associated with pulmonary hypertension [581], suggesting that a common underlying mechanism that affects the cardiopulmonary-renal axis may be implicated in sickle nephropathy and pulmonary hypertension. We have identified kidney injury molecule-1 (KIM-1) and N-acetyl-b-D-glucosaminidase (NAG) as two potential biomarkers of early SN [575] that need to be confirmed in longitudinal studies as true biomarkers of SN.

#### 11.1.6. Future Directions

SN has remained relatively underdiagnosed and understudied despite its onset early on in life: classic biomarkers of renal damage are normal even in the face of existing chronic sickle kidney disease, until extensive renal damage has occurred. Supranormal creatinine excretion in the urine results in lower than normal serum creatinine; therefore serum creatinine rises only in late stages of SN. Patients also have higher than normal renal blood flow and GFR; therefore urinary creatinine clearance is high from the resulting hyperfiltration, and subnormal GFR develops only when significant proteinuria and glomerular damage has developed and is largely irreversible [582]. Hence the proportion of patients diagnosed with SN only represents the tip of the iceberg; consequently, early interventions that would prevent the progression of renal damage cannot be applied. Second, the current paradigm remains that the hypoxic hyperosmotic medullary environment promotes sickling, causing progressive damage to the vasa recta, tubules, and nephrons over time. However, no formal studies have been performed on the molecular basis of SN. Third, not all patients with SCA develop SN, and it is not clear what factors predict or promote the progression of SN in susceptible patients. Finally, despite the known cumulative and progressive nature of organ damage in SCD, and the onset of SN as early as infancy, all current knowledge of SN is largely derived from cross-sectional analysis. There are no systematic longitudinal studies to characterize its natural history or progression.

## 12. Priapism

### 12.1. Pathophysiology

Priapism is a persistent unwanted and recurrent painful erectile erection that may last from hours to days. It is diagnosed by the provider based on patient self-report. During an episode, priapism can be confirmed by physical examination of finding a fully erect penis and complaint of pain in the penis and/or scrotum. There are no controlled studies on this complication of SCD. Most reports in the literature are case reports or observational studies [583–585]. Pathophysiologic mechanisms are not well understood and seem to pertain to a combination of hypoxia and impaired penile venous blood flow [586, 587]. The decreased rate of blood flow through the penis during normal erection allows increased oxygen extraction. As a result hypoxia promotes sickling with consequent congestion of the corpora, sludging, further impairment of venous outflow, and worsening hypoxia. Venous outflow from the corpora is reduced, and blood aspirated from the corpora during an episode of priapism is dark and has low pO2 and low glucose level [588].

### 12.2. Precipitating and Risk Factors

About 75% of priapism occurs between midnight and 6 AM and after sexual intercourse [586, 589, 590]. Acidosis resulting from dehydration and hypoventilation during sleep may be precipitating factors. Priapism affects 35% of boys and men [588]. Sexual intercourse [591], masturbation [592], alcohol intake [593], infection of the prostate or bladder, recent trauma, and medications with autonomic side effects are reported precipitating factors [594]. In the Jamaican study 16% of patients reported attacks following intercourse [595, 596]. Most episodes of priapism, however, have no obvious etiology.

Thrombocytosis, low level of Hb F, and severity of hemolysis are reported risk factors of priapism [586, 597, 598]. Recent studies have linked the severity of hemolysis to priapism, leg ulcers, and pulmonary hypertension [597]. These associations, however, have been challenged by other investigators [599].

### 12.3. Classification

Priapism has been classified in a number of ways [600]. Etiologically it could be idiopathic with no obvious underlying cause or secondary to trauma, infection, neoplasm, or hematologic disease including SCD, sickle trait, other hemoglobinopathies, polycythemia, other hemolytic disorders including enzymopathies and membranopathies and hematologic malignancies. Clinically priapism could be stuttering, minor, or major. Stuttering priapism is the occurrence of short, repetitive, and reversible painful episodes with detumescence occurring within a few hours after the onset of erection. This pattern has good prognosis and is associated with normal sexual function and rarely requires medical intervention. The prevalence of stuttering priapism varies from about 2% of men with SCD [601] to 40–60% of men with SCD according to other investigators [586, 596]. Minor priapism is isolated and infrequent episodes of painful erection that last less than 4 hours and do not require medical intervention. Major priapism, by contrast, is a prolonged episode of painful erection lasting longer than 12 hours that often requires hospitalization with medical and/or surgical intervention as described below. Partial or total impotence is often associated with major episodes of priapism. Anatomically, priapism could be bicorporal or tricorporal. Magnetic resonance imaging (MRI) of the penis can differentiate these two patterns. Bicorporal priapism involves both corpora cavernosa and is common in children with stuttering pattern. Tricorporal priapism involves both corpora cavernosa and the corpus spongiosum and is more common in older patients. It is painful erection that may last several days or weeks and may be followed by complete or partial impotence. Its prevalence varies between 6.5% [601] and 38% [586] of men with SCD. Stroke, chronic lung disease, chronic renal failure, and chronic leg ulcers were observed more frequently in men who had tricorporal priapism [601]. Priapism in adult males with SCD seems to be a marker of severe disease and identifies patients who are at risk for other sickle cell-related organ failures syndrome [597, 598].

### 12.4. Management

Major goals of management of priapism include pain relief and prevention of impotence. Minor episodes of priapism and stuttering priapism usually last less than 4 hours and are often treated at home with analgesics, benzodiazepines, or pseudoephedrine and do not require treatment in the emergency department (ED) or hospital. Patients are advised to report to the emergency department if an episode lasts longer than 4 hours. Initial treatment in the ED should include hydration and opioid analgesics. Catheterization of the urinary bladder may be indicated to promote emptying. If these measures fail to cause detumescence, penile aspiration and epinephrine irrigation should be utilized. Mantadakis et al. [602] recommend that aspiration of blood from the corpora cavernosa followed by irrigation with dilute epinephrine should be the initial therapy employed for patients with sickle cell anemia and prolonged priapism. This approach may be used by selected patients for self-management at home with aspiration and irrigation with dilute epinephrine solution. Simple transfusion or exchange transfusion may be tried for patients whose priapism does not respond to the aspiration and irrigation procedures and persists for 24 hours or longer [589, 590, 603]. Transfusion permits the entry of normal RBC to the engorged area and enhances oxygenation and improves blood flow. Siegel et al. [604], however, reported significant neurological complications in patients with priapism who had exchange transfusion. Analysis of their study shows that the Hb level after the blood exchange was much higher than the patient's baseline level. Thus the neurological complications were most likely due to the transfusion-induced hyperviscosity. A larger study using blood exchange transfusion for patients with priapism and keeping the postexchange Hb level similar to the baseline values showed no neurological complication in any of their patients [605]. Patients responding to transfusion therapy usually experience detumescence within 24 to 48 hours after the procedure. If detumescense does not occur within 24 hours following the completion of blood exchange transfusion, surgical intervention should be considered. Surgical intervention includes various shunt procedures between the cavernosa and the spongiosum [587, 606]. Without intervention, severe priapism results in impotence in more than 80% of patients. The combination of transfusions and surgery can decrease this to 25–50%. Patients who become impotent may benefit from psychological counseling and the insertion of prosthetic penile implants.

The role of hydroxyurea, etilefrine, leuprolide, sildenafil, and pseudoephedrine in preventing priapism is not well defined at the present [598]. Anecdotally hydroxyurea prevents or decreases the frequency of priapism in some patients.

## 13. Transfusion and Iron Overload

Blood transfusion is one of the three current major approaches for the effective and promising therapeutic approaches for SCD in general and SS in particular. The other two include hydroxycarbamide (HCD), also known as hydroxyurea (HU), and stem cell transplantation. Moreover blood transfusion could be symptomatic management of severe anemia or preventative management to prevent the occurrence of primary and secondary strokes in children with SS and the recurrence of acute chest syndrome in some patients. Historically blood transfusion was second to analgesia as a nonsurgical therapeutic approach for SS as far back as to the 1930s and 1940s even before the pathophysiology of the disease was well understood [607, 608]. Its utilization, however, followed a sinusoidal pattern over the years from underutilization to overutilization and back to what seems to be rational utilization at the present as will be mentioned below.

Because Hb S has decreased oxygen affinity and, hence, is efficient in delivering oxygen to tissues, most patients tolerate chronic anemia with an average Hb level of 7-8 g/dL well without the need for blood transfusion in the steady state [609, 610]. Nevertheless patient with SCD in general and patients with sickle cell anemia in particular are heavy utilizers of blood transfusion in part due to occasional exacerbation of the anemia and in part due to coexistent organ failure and tissue damage. Symptomatic anemia and a decrease in Hb level to values < 5 g/dL often necessitate blood transfusion. Jehovah's Witnesses, however, tolerate severe anemia with Hb level down to 2.7 g/dL [611]. Moreover, there is a direct relation between the Hb level in the steady state and the frequency of acute painful crises. Thus the relatively increased Hb level associated with mild anemia increases the blood viscosity that could precipitate acute painful episodes [612, 613]. Other vaso-occlusive episodes such as avascular necrosis are also more frequent in patients with mild anemia [614, 615]. Upon deoxygenation, the viscosity of blood containing significant amounts of Hb S rises sharply due to the polymerization of deoxy-HbS. A 50% increase in blood viscosity increases total peripheral resistance by 75% and reduces flow unless the pressure rises to compensate [616].

The transfusion of blood from normal donors to patients with sickle cell disease achieves the following two major goals: (1) improvement of the oxygen-carrying capacity of blood and its delivery to tissues and (2) dilution of circulating sickled red cells in order to improve microvascular perfusion. The achievement of these goals, however, is best if Hb S is decreased to <30% and the total Hb level is not >10 g/dL [617, 618]. Higher values of these parameters increase blood viscosity and its associated vaso-occlusive potential thus offsetting the benefits of blood transfusion.

The availability of safe blood due to advances in donor selection, the utilization of phenotype matching, the availability of oral iron chelators, and the reported efficacy of blood transfusion in preventing strokes and acute chest syndrome encouraged providers to use blood transfusion more frequently in managing the acute and chronic complications of SCD. Thus one study from a single institution found that about 50% of the patients with SS admitted to the hospital over 12-year period (1987–1998) received blood transfusion [619]. Another study also from a single institution [620] reported an increase in the % of transfused patients with SCD from 17.6% in 2000-2001 to 23.9% in 2008-2009.

The indications for blood transfusion have been classified in a number of ways. Moreover blood transfusions could be episodic or chronic, and the transfusion method could be simple transfusion or exchange transfusion [621–623]. In this paper indications for blood transfusion will be classified according to the desired objective of the transfusion. Thus the objectives of blood transfusion could be preventative/prophylactic, abortive/curative of acute complications, perioperative, symptomatic or for controversial objectives (Table 1). The logistic complexities and cost associated with blood exchange transfusion questioned whether simple chronic transfusion obviates the need for blood exchange transfusion. There is no easy answer to this query. The choice depends on the nature of the complications being treated. In acute complications such as acute chest syndrome the goal is to bring Hb S < 30% and the total Hb to 9-10 g/dL as soon as possible. If the patient in question has Hb level of 5 g/dL or less simple transfusion with 3-4 unit of RBC, to achieve the desired goal would be the appropriate choice. If the patient in question already has Hb level of 9 g/dL with Hb S >50%, blood exchange transfusion would be the appropriate and safe procedure to do. Moreover chronic simple transfusion will inevitably be associated with iron overload over a short period whereas chronic blood exchange transfusion may delay the onset of iron overload but is more likely to be associated with higher incidence of alloimmunization [228] unless extensive and expensive phenotype matching is utilized. The best approach seems to individualize the choice of the method of transfusion depending on the details of the clinical picture of the patient in question.

Blood selected for transfusion to patients with SCD should be sickle cell negative, ABO and Rh compatible, phenotypically matched for C, E, and K antigens, leuko-reduced and irradiated for selected patients [621]. Moreover washed RBC may be required in patients with severe allergic reactions due to plasma proteins. Fresh blood (blood less ≤ 10 days) is often requested for blood exchange transfusion although there is no evidence to support this request. Because the majority of patients with SCD are Africans or African Americans and because they receive blood given by Caucasians, there is high incidence of alloimmunization in transfused patients with SCD [621]. The most prevalent alloantibodies in patients with SS include anti-C, -E, and -K. Accordingly, the use of phenotypically matched blood, at least for these antigens, is highly recommended [621] and is currently routine practice in most sickle cell centers. Some blood banks implemented programs to increase communication between the African-American community and medical facilities to ensure the presence of blood supply from African-American donors directed for patients with SCD to reduce the incidence of alloimmunization to those antigens that are prevalent in African Americans. One concern about this practice of designated donations from African-Americans donors to patients with SCD is that it may increase the incidence of transfusion-related graft versus host disease unless such blood is routinely irradiated.

Complications of blood transfusion in patients with SCD include the transmission of infectious disease, alloimmunization, hemolytic transfusion reactions (acute or delayed), allergic reactions, febrile reactions, volume overload, and iron overload. Multiply transfused patients before 1992, when a screening test for hepatitis C was introduced in blood banks, were at risk for the transmission of hepatitis C virus (HCV). In one study [624] antibody to HCV (anti-HCV) was detected in 21% of patients with SS. Of the patients who received more than 10 units of blood, 30% were anti-HCV seropositive, whereas 9% of those patients who received <10 units were seropositive. Leukocyte-reduced RBC preparations are currently routinely used in most blood banks for blood transfusion at large. Leukocyte-reduced components reduce febrile reactions, decrease the chances for alloimmunization to leukocyte antigens, and minimize the transmission cytomegalovirus (CMV). Patients with SCD who are potential candidates for bone marrow or stem cell transplantation should receive irradiated cellular blood components.

On a brighter note chronic transfusion therapy is associated with a number of unintended benefits. These include decrease in the number of hospitalizations per year, decrease in the frequency of painful crises, and decrease in the frequency of acute chest syndrome [481].

Patients with SCD who receive blood transfusion chronically are at risk to develop iron overload. An observational study reported increased frequency of painful crises, organ damage and mortality in patients with SS and iron overload [619]. For billing purposes iron overload is coded under transfusional hemosiderosis or hemochromatosis. The total amount of body iron in an adult is about 3 g in women and 4 g in men. About 75% of body iron is in RBC, about one g in the liver and smaller trace amounts in myoglobin and certain enzymes. The human body has no mechanism to get rid of surplus body iron. Whatever iron is introduced into the body via infusion, blood transfusion or excessive oral intake remains in the body. The only methods to get rid of surplus body iron include bleeding, phlebotomy, or iron chelation therapy. One mL of RBC contains about one mg of iron, and the transfusion of one unit of RBC containing about 200 mL of RBC will introduce 200 mg of iron into the body. By the time 20 units of RBC are transfused, the total amount of iron introduced into the body will be about 4 g which is double the normal amount of total body iron. Thus the transfusion of 20 or more units of blood, by definition, is associated with iron overload provided there is no concomitant bleeding or phlebotomy.

The diagnosis of iron overload entails documentation of history of transfusion of ≥20 units of RBC in adults or ≥200 mL of RBC/kg in children and confirmation of iron overload by certain blood tests. These tests include serum ferritin level, liver biopsy to measure the amount of iron per g of liver dry weight and Ferriscan [625]. Other specific tests such as MRI 2* or Superconducting Quantum Interference Device (SQUID) are not available for general use. Iron overload is best monitored by periodic determinations of serum ferritin levels and transferrin saturation in frequently transfused patients in the steady state [619]. Ferritin is an acute phase reactant, and, hence, it could be falsely elevated during painful crises, infection or inflammation. To be a reliable indicator of iron overload, ferritin should be determined in the steady state at least on 3 separate occasions. If such measurements are consistent within a reasonable range of variation, they could confirm or deny the diagnosis of iron overload. Serum ferritin levels >1000 *μ*g/L and transferrin saturation >50% in the steady state are suggestive of iron overload. To further confirm the diagnosis of iron overload, a liver biopsy may be indicated. Hepatic iron concentration >7 mg/g liver dry weight is diagnostic of iron overload and is an indication for iron chelation therapy [626]. Ferriscan has been approved by the FDA, and its advantages are that it is a noninvasive and reliable test for the measurement of liver iron content. Its disadvantages include cost, length of time required to do the test, and its limitation to measure iron content in the liver only and not in other organs.

Three iron chelators are available for the therapeutic management of transfusional hemosiderosis [620, 627, 628]. These are deferoxamine (DFO), deferiprone (DFP), and deferasirox (DEFRA). DFP is not approved by the FDA in the USA and is not approved in Canada but is licensed in 61 other countries. Although DFO is an effective iron chelator that stood the test of time with over 40 years of experience, its utilization requires tedious and lengthy subcutaneous infusions over 10–12 hours daily for a minimum of 5 days per week. Compliance in children has been adequate but compliance in adults with SCD has been dismal. Oral DFP alone or in combination with DFO has been reported to be beneficial in general and for the management of cardiac iron overload in particular [629–632]. Major side effects of DFP include joint pain and joint swelling in 10–30% of patients, zinc deficiency in diabetic patients, and most seriously transient agranulocytosis in about 0.5–1.0% of patients usually during the first year of therapy associated with rare fatality. DEFRA is an oral chelator approved by the FDA with a starting daily dose of 20 mg/kg with gradual escalation of the dose if needed up to a maximum of 40 mg/kg/day [627]. The most common adverse effects of DEFRA included mild-to-severe skin rashes, mild gastrointestinal disturbances, and mild nonprogressive increase in serum creatinine and liver enzyme levels. Agranulocytosis, arthropathy, or growth failure due to DEFRA have not been documented to date. Nevertheless renal failure and bleeding with occasional fatalities have been reported. Needless to say the utilization of any of these chelators entails frequent and careful monitoring biweekly during the first two months of therapy and monthly thereafter. Recent review of clinical trials utilizing any of these chelators alone or in various combinations suggests that the use of chelation treatment in SCD to date has been based on little efficacy, and safety evidence although it is widely used and recommended [628]. More randomized clinical trials are needed to explore the safety, efficacy, and cost/benefit ratio evidence of iron chelation therapy in SCD.

## Figures and Tables

**Table 1:** Approaches to the management of sickle cell disease and its complications.

Approach	Definition
(1) Supportive management	Management intended to maintain the essential requirements for good health such as balanced diet, sleep, hydration, and folic acid

(2) Symptomatic management	Management targeted to alleviate the symptoms of the disease as they occur. These include blood transfusion for symptomatic anemia, analgesics for pain, and antibiotics for infections

(3) Preventative management	Approaches to prevent the occurrence of complications of the disease. These include things like vaccination, avoidance of stressful situations, Hb F induction with hydroxyurea or other agents, and transfusion to prevent the recurrence of stroke

(4) Abortive management	Major purpose of this approach is to abort painful crisis thus preventing them from getting worse or precipitating other complication. The only promising abortive approach has been nitric oxide

(5) Curative therapy	This is the ultimate goal of all inherited disorders. This has already been achieved in SCD by stem cell transplantation. Gene therapy is another challenging goal

**Table 2:** Staging of proliferative sickle retinopathy.

	Clinical findings
Stage I	Peripheral arteriolar occlusions

Stage II	Vascular remodeling, formation of arteriovenous anastomoses

Stage III	Peripheral retinal neovascularization

Stage IV	Vitreous hemorrhage

Stage V	Retinal detachment

**Table 3:** Principles of management of acute sickle cell pain.

(i) Assessment	
(ii) Choice of type of analgesic/dose/route	
(iii) Titration to relief/adjuvants	
(iv) Maintenance	
(v) Identify and treat side effects	
(vi) Adjustment for tolerance/rotation	
(vii) Tapering	
(viii) Switch to oral analgesics	
(ix) Specify disposition	

**Table 4:** Nonpharmacologic management of pain.

(i) TENS	(i) Relaxation
(ii) Vibration	(ii) Virtual reality
(iii) Massage	(iii) Meditation
(iv) Heat or ice packs	(iv) Self-hypnosis
(v) Menthol rub	(v) Acupressure
(vi) Therapeutic exercise	(vi) Acupuncture
(vii) Music	(vii) Biofeedback

**Table 5:** Classification of opioid analgesicsg.

(1) *μ* Agonists	
Codeine: O, P	
Hydrocodone: O	
Morphine: O, P, IR, CR	
Hydromorphone: O, P, IR, CR	
Oxycodone: O, IR, CR	
Meperidine: O, P, IR	
Fentanyl: TD, OTM, OBU, P, IR, CR	
Oxymorphone: O, P, IR, CR	
Methadone: O, P, LA	
Levorphanol: O, P, LA	
(2) Partial agonists	
Buprenorphine: P, OSLG, TD	
(3) Mixed agonists antagonists	
Pentazocine: O, P, IR	
Nalbuphine: P, IR	
Butorphanol: P, IN, IR	
(4) Others	
Tapentadol: O, IR	
Tramadol: O, IR, CR	

O; oral; P; parenteral; IR; immediate release; CR; controlled release; TD; transdermal; OTM; oral transmembranous; OBU; oral buccal; LA; long-acting; OSLG; oral sublingual; IN; intranasal.
